# Azaphilones from the Red Sea Fungus *Aspergillus falconensis*

**DOI:** 10.3390/md18040204

**Published:** 2020-04-10

**Authors:** Dina H. El-Kashef, Fadia S. Youssef, Rudolf Hartmann, Tim-Oliver Knedel, Christoph Janiak, Wenhan Lin, Irene Reimche, Nicole Teusch, Zhen Liu, Peter Proksch

**Affiliations:** 1 Institute of Pharmaceutical Biology and Biotechnology, Heinrich-Heine-University Duesseldorf, 40225 Duesseldorf, Germany; dina.elkashef@mu.edu.eg (D.H.E.-K.); fadiayoussef@pharma.asu.edu.eg (F.S.Y.); 2 Department of Pharmacognosy, Faculty of Pharmacy, Minia University, 61519 Minia, Egypt; 3 Department of Pharmacognosy, Faculty of Pharmacy, Ain Shams University, Abbassia, 11566 Cairo, Egypt; 4 Institute of Complex Systems: Strukturbiochemie, Forschungszentrum Jülich GmbH, ICS-6, 52425 Jülich, Germany; r.hartmann@fz-juelich.de; 5 Institut für Anorganische Chemie und Strukturchemie, Heinrich-Heine-Universität Düsseldorf, 40225 Düsseldorf, Germany; tim-oliver.knedel@hhu.de (T.-O.K.); janiak@uni-duesseldorf.de (C.J.); 6 State Key Laboratory of Natural and Biomimetic Drugs, Peking University, Beijing 100191, China; whlin@bjmu.edu.cn; 7 Department of Biomedical Sciences, Institute of Health Research and Education, University of Osnabrück, 49074 Osnabrück, Germany; irene.reimche@uni-osnabrueck.de (I.R.); nicole.teusch@uni-osnabrueck.de (N.T.); 8 Hubei Key Laboratory of Natural Products Research and Development, College of Biological and Pharmaceutical Sciences, China Three Gorges University, Yichang 443002, China

**Keywords:** *Aspergillus falconensis*, OSMAC, azaphilones, X-ray diffraction, NF-κB inhibition

## Abstract

The marine-derived fungus *Aspergillus falconensis*, isolated from sediment collected from the Canyon at Dahab, Red Sea, yielded two new chlorinated azaphilones, falconensins O and P (**1** and **2**) in addition to four known azaphilone derivatives (**3**−**6**) following fermentation of the fungus on solid rice medium containing 3.5% NaCl. Replacing NaCl with 3.5% NaBr induced accumulation of three additional new azaphilones, falconensins Q−S (**7**−**9**) including two brominated derivatives (**7** and **8**) together with three known analogues (**10**−**12**). The structures of the new compounds were elucidated by 1D and 2D NMR spectroscopy and HRESIMS data as well as by comparison with the literature. The absolute configuration of the azaphilone derivatives was established based on single-crystal X-ray diffraction analysis of **5**, comparison of NMR data and optical rotations as well as on biogenetic considerations. Compounds **1**, **3**−**9**, and **11** showed NF-κB inhibitory activity against the triple negative breast cancer cell line MDA-MB-231 with IC_50_ values ranging from 11.9 to 72.0 µM.

## 1. Introduction

In the last two decades, marine-derived fungi have gained considerable attention for drug discovery due to their ability to produce a vast diversity of bioactive secondary metabolites [1]. Up until today, hundreds of secondary metabolites have been characterized from marine-derived fungi exhibiting promising biological and pharmacological properties [2,3]. In particular, the diketopiperazine alkaloid halimide, obtained from a marine-derived fungus *Aspergillus* sp., was the lead structure for the putative anticancer drug plinabulin, which has entered Phase III of clinical trials against non-small cell lung cancer [4,5,6]. Interestingly, genomic sequencing revealed that under conventional culture conditions, many biosynthetic fungal gene clusters remain silent and transcriptionally suppressed [7,8]. Changing the cultivation conditions of fungi may activate silent biosynthetic gene clusters and eventually lead to either upregulation of constitutively present compounds or accumulation of new natural products [9]. The OSMAC (One Strain MAny Compounds) approach, first described by Zeeck et al. [10], represents one of the strategies which triggers diversification of the metabolic profile of fungi by altering the cultivation conditions. Fungi of the genus *Aspergillus* are rich sources of numerous bioactive secondary metabolites [11,12,13]. Consequently, as a part of our ongoing research on marine-derived fungi [14,15,16], we have investigated the fungus *Aspergillus falconensis* (formerly known as *Emericella falconensis* [17]) that was isolated from sea sediment collected at a depth of 25 m from the Canyon at Dahab, Red Sea, Egypt. The previously isolated soil-derived fungus, *Emericella falconesis*, is known as a producer of anti-inflammatory azaphilone derivatives [18,19,20,21]. Herein, we report the isolation, structure elucidation, and bioactivity of azaphilones obtained from *A. falconensis* following fermentation of the fungus on solid rice medium that contained either 3.5% NaCl or 3.5% NaBr. Following this approach, we were able to obtain two new chlorinated azaphilones (**1** and **2**) together with four known azaphilone derivatives (**3**–**6**) when the fungus was cultivated in the presence of NaCl and three additional new azaphilone derivatives (**7**–**9**) including two brominated analogues (**7** and **8**) in addition to three known derivatives (**10**–**12**) in the presence of NaBr. Compounds **1**, **3**–**9**, and **11** were examined with regard to their nuclear factor kappa B (NF-κB) inhibitory activity in the triple negative breast cancer (TNBC) cell line MDA-MB-231. In TNBC, constitutive activation of the proinflammatory NF-κB is associated with tumor aggressiveness [22]. All tested compounds revealed inhibition of NF-κB signaling with IC_50_ values at two-digit micromolar concentrations.

## 2. Results and Discussion

After fermentation of *A. falconensis* on solid rice medium containing 3.5% NaCl (similar to the salinity of sea water), chromatographic separation of the EtOAc extract of the fungus yielded two new chlorinated azaphilone derivatives (**1**–**2**). In addition, four known azaphilones (**3**–**6**) were identified including falconensins A (**3**) [18], M (**4**) [23], N (**5**) [23], and H (**6**) [19] by comparison of their spectroscopic data with the literature.

Compound **1** was isolated as a yellow oil. Its HRESIMS analysis showed an isotope pattern characteristic for two chlorine atoms in the molecule at *m*/*z* 511/513/515 (9:6:1), corresponding to the molecular formula C_24_H_24_Cl_2_O_8_ with 12 degrees of unsaturation. The UV pattern and NMR data of **1** (Table 1 and Table 2) were similar to those of the co-isolated known falconensin M (**4**), which was previously reported from *Emericella falconensis* [23], suggesting **1** to be an azaphilone derivative [24]. However, the ^1^H NMR spectrum of **1** displayed the signal of an additional methyl group at *δ*_H_ 2.07 (s) when compared to **4**. The Heteronuclear Multiple Bond Correlation (HMBC) correlations from the protons of this additional methyl and H-8 to a carbonyl carbon at *δ*_C_ 170.0 together with the obvious deshielded chemical shift of H-8 (*δ*_H_ 6.11) indicated the replacement of the hydroxy group by an acetoxy group at C-8 in **1**. Detailed analysis of the 2D NMR spectra of **1** indicated that the remaining substructure of **1** was identical to that of **4**. The large values of ^3^*J*_H-8,H-8a_ (10.7 Hz) and ^3^*J*_H-1b,H-8a_ (13.0 Hz) and the small value of ^3^*J*_H-1a,H-8a_ (5.0 Hz) obtained from the ^1^H NMR spectrum of **1**, indicated *trans*-diaxial orientation between H-8a (*δ*_H_ 2.98) and H-8 and between H-8a and H-1b (*δ*_H_ 3.96) and *cis*-orientation between H-8a and H-1a (*δ*_H_ 4.34). The NOE correlations from H-8a to H-1a and Me-9 (*δ*_H_ 1.56) as well as between H-8 and H-1b confirmed that H-1a, H-8a and Me-9 were on the same side of the ring (α-oriented) whereas H-1b and H-8 were on the opposite side (β-oriented). Thus, compound **1** was elucidated as 8-*O*-acetyl analogue of **4**, representing a new falconensin derivative, for which the trivial name falconensin O is proposed. Incubation of putative precursors of **1** such as **4** in EtOAc over three days failed to generate the corresponding acetates, thus ruling out the possibility that the acetylated azaphilones isolated in this study are artefacts generated during chromatographic workup.

The molecular formula of compound **2** was deduced as C_24_H_26_Cl_2_O_8_, containing two additional protons when compared to **1**. Investigation of the ^1^H and ^13^C NMR data of **2** (Table 1 and Table 2) demonstrated the close structural similarity between **1** and **2** except for signals of the side chain at C-3. In particular, the resonances for the olefinic methine protons at *δ*_H_ 5.91 (H-10) and 6.45 (H-11) of **1** were replaced by two methylene groups at *δ*_H_ 2.20 (H_2_-10) and 1.58 (H_2_-11) in **2**. The COSY correlations between H_2_-10/H_2_-11/Me-12 together with the HMBC correlations from H_2_-11 to C-3 (*δ*_C_ 168.2) and from H_2_-10 to C-3 and C-4 (*δ*_C_ 100.9) confirmed the attachment of a *n*-propyl moiety at C-3 in **2**. The remaining substructure of **2** was determined to be identical to that of **1** by comparison of the 2D NMR data of **1** and **2**. The similar coupling constants and NOE relationships between **1** and **2** indicated that both share the same relative configuration.

When 3.5% NaCl as a constituent of the rice medium was replaced with 3.5% NaBr, a profound change in the metabolic profile of the fungus was observed. In total, two new brominated azaphilone derivatives (**7** and **8**), one new non-halogenated azaphilone (**9**), in addition to the known falconensins K (**10**) [23] and I (**11**) [23] were obtained.

**Figure 1:**
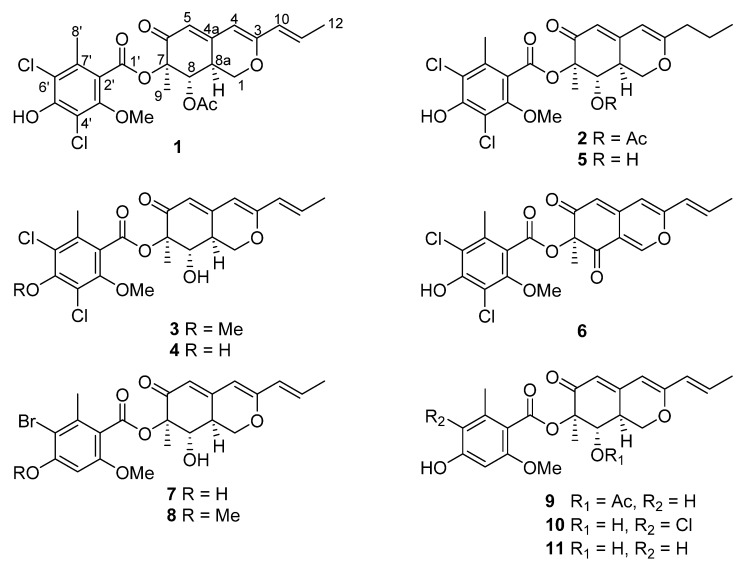
Structures of azaphilones isolated from *A. falconensis*.

The ^1^H and ^13^C NMR spectra of **7** (Table 1 and Table 2) were comparable to those of the co-isolated known compound, falconensin K (**10**) [23]. The HRESIMS data of **7** established the molecular formula C_22_H_23_BrO_7_, differing from that of **10** by the replacement of the chlorine with a bromine atom. The HMBC correlations from Me-8′ (*δ*_H_ 2.48) to C-2′ (*δ*_C_ 117.4), C-6′ (*δ*_C_ 105.3), and C-7′ (*δ*_C_ 137.4), from H-4′ to C-2′, C-6′, C-3′ (*δ*_C_ 156.2), and C-4′ (*δ*_C_ 154.0), from 3′-OMe (*δ*_H_ 3.83) to C-3′ together with the NOE correlation between 3′-OMe and Me-9 (*δ*_H_ 1.47) indicated the position of the bromine atom at C-6′ in **7.** The remaining azaphilone core structure including the relative configuration of **7** was confirmed to be identical to that of **10** after further inspection of the 2D NMR spectra of **7**. Thus, the structure of **7** was elucidated as shown (Figure 1), and the trivial name falconensin Q is proposed for this compound.

Falconensin R (**8**) was isolated as a yellow oil. Its molecular formula was determined as C_23_H_25_BrO_7_ by HRESIMS, containing an additional methyl group when compared to **7**. The NMR data of **8** (Table 1 and Table 2) were similar to those of **7** except for the appearance of two methoxy groups at *δ*_C_ 56.4 and 56.3, and at *δ*_H_ 3.89 (5′-OMe) and 3.92 (3′-OMe) instead of one methoxy group in **7**. The location of the additional methoxy group at C-5′ in **8** was confirmed by the HMBC correlations from 3′-OMe to C-3′ (*δ*_C_ 157.3), from 5′-OMe to C-5′ (*δ*_C_ 155.8), and from H-4′ to C-2′ (*δ*_C_ 117.2), C-6′ (*δ*_C_ 106.6), C-3′ and C-5′, as well as based on the NOE correlations from H-4′ to 3′-OMe and 5′-OMe.

The molecular formula of **9** was determined as C_24_H_26_O_8_ by HRESIMS, indicating 42 amu more than that of the co-isolated known falconensin I (**11**) (C_22_H_24_O_7_) [23]. Comparison of the ^1^H and ^13^C NMR spectra revealed that the structure of **9** was closely related to that of **11**, except for the appearance of an additional acetoxy group at *δ*_H_ 2.15 and *δ*_C_ 20.8 and 169.7 in **9**. The location of this additional acetoxy group at C-8 was confirmed by the COSY correlations between H-1ab/H-8a/H-8 and the HMBC correlations from H-8 to the carbon of the additional acetoxy group. The similar NOE correlations of **9** and **11** suggested that they shared the same configuration. Thus, compound **9** was elucidated as 8-*O*-acetyl analogue of falconensin I (**11**).

**Figure 2:**
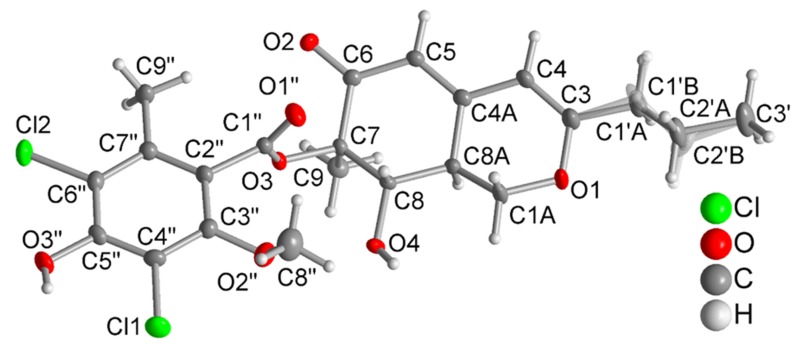
Molecular structure of **5** from a single-crystal X-ray structure determination (50% thermal ellipsoids; H atoms with arbitrary radii). The propyl group is disordered, and the two respective atom positions were refined with equal occupation factors.

The specific optical rotation values of **1**, **2**, **7**, **8**, and **9** are positive (+99 − +233), which is in agreement with the positive values reported for other known falconensin derivatives with 7*R*, 8*S*, and 8a*S* configuration, suggesting that the new compounds share the same absolute configuration as the known derivatives [18,20,25]. Although the absolute configuration of other falconensin derivatives had been determined previously by Mosher’s reaction and observation of Cotton effects of CD curves [18,20,25], no crystal structure had so far been reported for these compounds. To independently assign the absolute configuration of the isolated compounds, a single crystal X-ray diffraction analysis through anomalous dispersion of the known falconensin N (**5**), for which crystals of sufficient quality were obtained, was conducted. Herein, we present for the first time the crystal structure of falconensin N (**5**) (Figure 2). From the single-crystal structure refinement, the absolute structure assignment was based on the Flack parameter of 0.016(5) (Table S1) [26,27,28,29]. The crystal structure of falconensin N (**5**) is in agreement with the previously reported absolute configuration [18,20,25]. Based on these data, all falconensin derivatives isolated in this study are suggested to share the same 7*R*, 8*S*, and 8a*S* absolute configuration.

Azaphilones constitute a class of fungal metabolites possessing various biological activities, including antiviral, antibacterial, antioxidant, hypolipidemic, cytotoxic, and anti-inflammatory properties [24,30]. For falconensins in particular, anti-inflammatory activity against 12-*O*-tetradecanoylphorbol-13-acetate-induced inflammatory ear edema in mice had been reported [21]. Compounds **1**, **3**–**9**, and **11** were evaluated for their anti-inflammatory activity in the triple negative breast cancer cell line NF-κB-MDA-MB-231. The IC_50_ values were calculated based on inhibition of the NF-κB-dependent luciferase activity and revealed NF-κB blockade for all compounds (Table 3). To exclude that cytotoxicity caused reduction of NF-κB activity, cell viability was determined in parallel. Compounds **1**, **4**–**7**, **9**, and **11** did not influence cell viability within the selected concentration range, whereas compound **8** reduced cell viability with an IC_50_ of 126.8 ± 5.4 µM, thus showing about 9-times higher potency in NF-κB-blockade compared to its cytotoxicity. Compound **3** with an IC_50_ of 89.7 ± 9.1 µM in the cytotoxicity assay only showed around 2-times higher potency in NF-κB-blockade. Plotting the pIC_50_, which is calculated as the negative decadic logarithm of IC_50_, of the cell viability against the pIC_50_ of NF-κB inhibition, illustrates a greater anti-inflammatory potential of the examined compounds compared to their cytotoxic potential (Figure S40). In conclusion, inhibition of NF-κB signaling in the TNBC cell line MDA-MB-231 could be induced by **1**, **3** − **9**, and **11**, with **7** being the most potent compound.

## 3. Materials and Methods

### 3.1. General Experimental Procedures

Optical rotations were measured using a Jasco P-2000 polarimeter. The compounds were dissolved in optically pure solvents Uvasol^®^ (spectroscopic grade solvents, Merck). 1D and 2D NMR spectra were recorded on Bruker Avance III 300 or 600 or 700 MHz NMR spectrometers (Bruker BioSpin, Rheinstetten, Germany). Low-resolution mass spectra were recorded with an Ion-Trap-API Finnigan LCQ Deca (Thermo Quest) mass spectrometer, while high-resolution mass data were measured on a FTHRMS-Orbitrap (Thermo-Finnigan) mass spectrometer. HPLC analysis was conducted using a Dionex UltiMate-3400 SD with an LPG-3400SD pump coupled to a photodiode array detector (DAD3000RS) and employing a Knauer Eurospher C_18_ analytical column (125 × 4 mm i.d., 5 μm). Purification of the compounds was performed using semipreparative HPLC on the VWR Hitachi Chromaster HPLC system, 5160 Pump; 5410 UV detector; Eurosphere-100 C_18_, 300 mm × 8 mm i.d., 10 µm, Knauer, Germany) with MeOH and H2O as eluents utilizing a flow rate of 5 mL/min. Column chromatography was performed using different stationary phases including Merck MN silica gel 60 M (0.04−0.063 mm), silica gel 60 RP-18 (40–63 µm), and Sephadex LH-20 (Merck). TLC plates precoated with silica gel 60 F_254_ (Merck) were used for monitoring fractions resulting from column chromatography. Detection of spots on the TLC was done by UV absorption at 254 and 365 nm followed by anisaldehyde spray reagent.

### 3.2. Fungal Material

The fungus was isolated from marine sediment which was collected at a depth of 25 m from the Canyon at Dahab, Red Sea, Egypt in November 2016. The fungus was identified as *A. falconensis* (GenBank accession No. MN905375) through amplification and sequencing of the internal transcribed spacer region including the 5.8S ribosomal DNA following by a subsequent BLAST search in NCBI as described before [31]. A deep-frozen specimen of the fungal strain has been deposited in one of the author’s laboratory (P.P.).

### 3.3. Fermentation, Extraction, and Isolation

Initial fermentation of the fungus was conducted in ten 1L Erlenmeyer flasks. To each flask, 100 g of rice (Oryza Milchreis), 100 mL of demineralized water, and 3.5 g of NaCl were added. Thereafter, the flasks were autoclaved at 121 °C for 20 min and after cooling to room temperature, the fungus was inoculated on the rice medium. Fermentation of the fungus was continued under static conditions for 21 days at 20 ℃ until the fungus had totally overgrown the medium. Then, each flask was extracted with 600 mL EtOAc. After overnight soaking in EtOAc, the rice medium was cut into small pieces and shaken for 8 h at 150 rpm followed by evaporation of EtOAc, yielding around 16 g of EtOAc extract. For the OSMAC experiment, the same cultivation and extraction procedures of the initial cultivation were conducted except for the replacement of the added 3.5% NaCl with 3.5% NaBr and the cultivation on three flasks instead of ten. Eventually, the OSMAC experiment yielded approximately 2 g of EtOAc extract.

The initial crude extract of the large scale cultivation of the fungus obtained in presence of NaCl (16 g) was fractionated by vacuum liquid chromatography (VLC) on silica gel as a stationary phase utilizing a step gradient of solvents consisting of mixtures of *n*-hexane/EtOAc and CH_2_Cl_2_/MeOH, to yield 12 fractions (V1 to V12). Fraction V4 (874 mg) was further separated by Sephadex LH20 column chromatography using CH_2_Cl_2_-MeOH (1:1) as mobile phase affording eight subfractions (V4-S1 to V4-S8). Subfraction V4-S4 (162.8 mg) was purified by semi-preparative HPLC using acetonitrile-H_2_O containing 0.1% formic acid (from 60:40 to 95:5 in 22 min) to afford **1** (1.8 mg), **2** (1.5 mg), and **3** (11.8 mg). Subfraction V4-S5 (139.6 mg) was further purified by semi-preparative HPLC using gradient elution with acetonitrile-H_2_O containing 0.1% formic acid (60:40 to 95:5 in 22 min) affording **4** (6.3 mg) and **5** (2.2 mg). Fraction V6 (686.1 mg) was submitted to RP-VLC column using H_2_O-MeOH gradient elution to yield 10 subfractions (V6-R1 to V6-R10). Subfraction V6-R7 (110 mg) was further purified using semi-preparative HPLC with a gradient of MeOH-H_2_O containing 0.1% formic acid (67:33 to 80:20 in 20 min) to afford **6** (15.1 mg).

The EtOAc extract (2 g) obtained from the OSMAC experiment with addition of 3.5% NaBr, was also fractionated by VLC on silica gel as described before to give 13 fractions (BrV1 to BrV13). Subsequent purification of BrV5 (157 mg), BrV6 (32 mg), and BrV10 (140 mg) using Sephadex LH20 column chromatography with CH_2_Cl_2_-MeOH (1:1) as mobile phase and semi-preparative HPLC with a gradient of MeOH-H_2_O containing 0.1% formic acid to afford **7** (3.1 mg), **8** (2.1 mg), **9** (1.2 mg), **10** (0.9 mg), and **11** (2.0 mg).

*Falconensin O* (**1**): Yellow oil; ${\left[\alpha \right]}_{D}^{20}$ +176 (*c* 0.2, MeOH); UV (MeOH) λ_max_ 355 and 206 nm; ^1^H and ^13^C NMR data, Table 1 and Table 2; HRESIMS *m*/*z* 511.0923 [M + H]^+^ (calcd for C_24_H_25_Cl_2_O_8_, 511.0921).

*Falconensin P* (**2**): Yellow amorphous powder; ${\left[\alpha \right]}_{D}^{20}$ +233 (*c* 0.2, MeOH); UV (MeOH) λ_max_ 329 and 216 nm; ^1^H and ^13^C NMR data, Table 1 and Table 2; HRESIMS *m*/*z* 513.1073 [M + H]^+^ (calcd for C_24_H_27_Cl_2_O_8_, 513.1077).

*Falconensin Q* (**7**): Yellow oil; ${\left[\alpha \right]}_{D}^{20}$ +182 (*c* 0.2, MeOH); UV (MeOH) λ_max_ 354 and 205 nm; ^1^H and ^13^C NMR data, Table 1 and Table 2; HRESIMS *m*/*z* 479.0702 [M + H]^+^ (calcd for C_22_H_24_BrO_7_, 479.0700). 

*Falconensin R* (**8**): Yellow oil; ${\left[\alpha \right]}_{D}^{20}$ +99 (*c* 0.2, MeOH); UV (MeOH) λ_max_ 353 and 206 nm; ^1^H and ^13^C NMR data, Table 1 and Table 2; HRESIMS *m*/*z* 493.0853 [M + H]^+^ (calcd for C_23_H_26_BrO_7_, 493.0856).

*Falconensin S* (**9**): Yellow oil; ${\left[\alpha \right]}_{D}^{20}$ +105 (*c* 0.2, MeOH); UV (MeOH) λ_max_ 355 and 201 nm; ^1^H and ^13^C NMR data, Table 1 and Table 2; HRESIMS *m*/*z* 443.1700 [M + H]^+^ (calcd for C_24_H_27_O_8_, 443.1700).

### 3.4. Crystallographic Analysis of Compound ***5***

Crystals were obtained by solvent evaporation (MeOH). Data Collection: compound **5** was measured with a Bruker Kappa APEX2 CCD diffractometer with a microfocus tube and Cu-Kα radiation (λ = 1.54178 Å). APEX2 was used for data collection, SAINT for cell refinement and data reduction [32], and SADABS for experimental absorption correction [33]. The structure was solved by intrinsic phasing using SHELXT [34], and refinement was done by full-matrix least-squares on F^2^ using SHELXL-2016/6 [35]. The hydrogen atoms were positioned geometrically (with C-H = 0.95 Å for aromatic CH, 1.00 Å for aliphatic CH, 0.99 Å for CH_2_, and 0.98 Å for CH) and refined using riding models (AFIX 43, 13, 23, 137, respectively), with Uiso(H) = 1.2 Ueq(CH, CH_2_), and 1.5 Ueq(CH_3_). The absolute structure configuration of **5** was solved using anomalous dispersion from Cu-Kα, resulting in a Flack parameter of 0.016(5) using Parsons quotient method. All graphics were drawn using DIAMOND [36]. The structural data have been deposited in the Cambridge Crystallographic Data Center (CCDC No. 1976223).

### 3.5. Triple Negative Breast Cancer Studies

#### 3.5.1. Cell Culture and Chemicals

Culture medium and supplements were purchased from Gibco (Fisher Scientific, Schwerte, Germany). Cell plates were obtained from Greiner bio-one (Frickenhausen, Germany). Cells were grown and incubated in a humidified 5% CO_2_ atmosphere at constant 37 °C. The metastatic breast cancer cell line, MDA-MB-231, was purchased from the European Collection of Authenticated Cell Cultures (ECACC) (Salisbury, UK). Sub-culturing was performed in RPMI 1640 medium (#21875-034) supplemented with 15% (v/v) fetal calf serum (FCS) and 1% (v/v) penicillin-streptomycin (pen-strep) (10,000 U/mL). The MDA-MB-231 cell line stably expressing a plasmid with the NF-κB response element and the gene sequence for the firefly-luciferase protein (NF-κB- MDA-MB-231) was purchased from Signosis (Santa Clara, CA, USA; #SL-0043). For selection, 100 µg/mL hygromycin B (Life Technologies, Darmstadt, Germany; #10687010) was applied in high glucose DMEM (#41966-029) supplemented with 10% (v/v) FCS, 1% (v/v) pen-strep. Starvation medium for the NF-κB inhibition assay contained 1% (v/v) FCS, 1% (v/v) pen-strep, and 100 µg/mL hygromycin B. Cell detachment occurred by trypsinization in 0.25% trypsin-EDTA and cell counting was performed at 1:1 (v/v) dilution in Erythrosin B (BioCat, Heidelberg, Germany; #L13002) using the LUNA II automated cell counter (BioCat). Compounds **1**, **4**, **5**, **7**, **8**, and **9** were dissolved in dimethyl sulfoxide (DMSO) to a final concentration of 10 mM, whereas the compounds **3**, **6**, **11** were dissolved at 20 mM in DMSO. Further dilutions in cell culture medium contained maximal 2% DMSO.

#### 3.5.2. NF-κB Inhibitory Assay

3 × 10^4^ NFκB-MDA-MB-231 cells were seeded in total 100 µL medium per well in a 96-well plate (Greiner; #655098). On the next day, medium was exchanged, and triplicates were pre-incubated for 20 min without (negative control) or with compounds in total 100 µL starvation medium. Final concentration of the compounds ranged a twofold serial dilution starting from 400 µM (compound **3**, **6**, and **11**) or 200 µM (compound **1**, **4**, **5**, **7**, **8**, and **9**) to 0.78 µM. To activate NF-κB signaling, untreated cells (positive control) or compound treated cells were subsequently stimulated for 24 h with 20 ng/mL TNFα (Peprotech, Hamburg, Germany; #300-01A). Finally, cell lysis and measurement were done according to the manufacturer’s instruction of the Luciferase Assay System (Promega, Mannheim, Germany; #E1500). Injection of equal volume of luciferase substrate with 10 s integration time and subsequent luminescence measurement was performed using the Spark^®^ microplate reader (TECAN, Männedorf, Switzerland).

#### 3.5.3. Cell Viability Assay

Using the CyBio^®^ Well vario pipetting robot (Analytik Jena, Jena, Germany; #OL3381-24-730), 18 µL of the MDA-MB-231 cell suspension (2.8 × 10^5^ cells/mL) were seeded per well in a 384-well plate (Greiner; #781074) and incubated for 24 h. The final concentration of the compounds ranged in twofold serial dilution steps starting from 400 µM (compound **3**, **6**, and **11**) or 200 µM (compound **1**, **4**, **5**, **7**, **8**, and **9**) to 0.78 µM. After 24 h compound stimulation in quadruples, cell lysis and measurement were done as prescribed in the manufacturer’s instruction of the CellTiter-Glo^®^ Luminescent Cell Viability Assay (Promega; #G7570). In short, it was applied equal volume of the CellTiter-Glo^®^ reagent and luminescence was measured using the Spark^®^ microplate reader (TECAN).

#### 3.5.4. Statistical Analysis

Data of the NF-κB inhibitory assay and cell viability assay represent at least three individual experiments and were analyzed using GraphPad Prism (GraphPad Software, San Diego, USA; Version 8.1.2). For the NF-κB inhibitory assay, data below the relative light unit (RLU) of the negative control were excluded for further analysis. Half maximal inhibitory concentration (IC_50_) values were determined by nonlinear regression analysis based on the dose–response inhibition calculation “log(inhibitor) vs. response–variable slope (four parameters)” without curve fitting.

## Figures and Tables

**Table 1:** ^13^C NMR data of compounds **1**, **2**, **7**, **8**, and **9** in CDCl_3_.

No.	1 ^a,^^c^	2 ^a,^^c^	7 ^b^	8 ^a,^^c^	9 ^b^
1	67.8, CH_2_	68.2, CH_2_	68.5, CH_2_	68.4, CH_2_	68.0, CH_2_
3	160.2, C	168.2, C	160.5, C	160.2, C	160.2, C
4	102.4, CH	100.9, CH	102.8, CH	102.6 CH	102.8, CH
4a	149.4, C	149.2, C	150.3, C	149.9, C	148.9, C
5	116.6, CH	115.7, CH	116.6, CH	116.6, CH	117.1, CH
6	192.3, C	192.7, C	193.8, C	193.4, C	193.3, C
7	83.4, C	83.4, C	85.6, C	85.4, C	82.5, C
8	70.1, CH	70.4, CH	69.8, CH	69.7, CH	70.7, CH
8a	38.1, CH	38.0, CH	37.8, CH	37.5, CH	38.2, CH
9	17.9, CH_3_	18.2, CH_3_	16.8, CH_3_	16.6, CH_3_	18.2, CH_3_
10	124.9, CH	36.4, CH_2_	125.4, CH	125.3, CH	125.2, CH
11	134.1, CH	20.0, CH_2_	133.9, CH	133.6, CH	133.9, CH
12	18.2, CH_3_	13.6, CH_3_	18.4, CH_3_	18.2, CH_3_	18.4, CH_3_
1′	164.3, C	164.4, C	165.3, C	164.9, C	166.2, C
2′	122.2, C	122.3, C	117.4, C	117.2, C	115.5, C
3′	152.8, C	152.9, C	156.2, C	157.3, C	159.0, C
4′	112.9, C	112.8, C	97.1, CH	93.8, CH	96.9, CH
5′	149.2, C	149.8, C	154.0, C	155.8, C	157.6, C
6′	117.0, C	117.1, C	105.3, C	106.6, C	109.2, CH
7′	134.3, C	134.5, C	137.4, C	138.1, C	140.0, C
8′	16.9, CH_3_	17.1, CH_3_	20.2, CH_3_	20.1, CH_3_	19.6, CH_3_
8-OAc	170.0, C	170.1, C			169.7, C
	20.5, CH_3_	20.8, CH_3_			20.8, CH_3_
3′-OMe	62.4, CH_3_	62.6, CH_3_	56.5, CH_3_	56.3, ^d^ CH_3_	56.0, CH_3_
5′-OMe				56.4, ^d^ CH_3_	

^a^ Measured at 150 MHz; ^b^ Measured at 175 MHz; ^c^ Data extracted from the HSQC (Heteronuclear Single Quantum Coherence) and HMBC spectra; ^d^ interchangeable.

**Table 2:** ^1^H NMR data of compounds **1**, **2**, **7**, **8**, and **9** in CDCl_3_.

No.	1 ^a^	2 ^a^	7 ^b^	8 ^a^	9 ^b^
1	4.34, dd (11.0, 5.0); 3.96, dd (13.0, 11.0)	4.29, dd (11.0, 5.1); 3.93, dd (13.2, 11.0)	4.77, dd (10.5, 5.1); 3.84, dd (13.0, 10.5)	4.77, dd (10.6, 5.0); 3.84, dd (13.0, 10.6)	4.32, dd (10.8, 5.0); 3.93, dd (13.0, 10.8)
4	5.59, s	5.53, s	5.56, s	5.57, s	5.57, s
5	5.86, d (1.7)	5.79, d (1.9)	5.80, d (1.6)	5.80, d (1.8)	5.85, d (1.9)
8	6.11, d (10.7)	6.09, d (10.7)	4.74, d (10.7)	4.75, d (10.7)	6.11, d (10.7)
8a	2.98, dddd (13.0, 10.7, 5.0, 1.7)	2.94, dddd (13.2, 10.7, 5.1, 1.9)	2.88, dddd (13.0, 10.7, 5.1, 1.6)	2.88, dddd (13.0, 10.7, 5.0, 1.8)	2.96, dddd (13.0, 10.7, 5.0, 1.9)
9	1.56, s	1.56, s	1.47, s	1.47, s	1.54, s
10	5.91, dq (15.4, 1.3)	2.20, m	5.90, dq (15.4, 1.4)	5.90, dd (15.4, 1.5)	5.90, dd (15.3, 1.6)
11	6.45, dq (15.4, 7.0)	1.58, m	6.47, dq (15.4, 7.0)	6.46, dd (15.4, 7.0)	6.42, dd (15.3, 7.0)
12	1.88, dd (7.0, 1.3)	0.94, t (7.4)	1.87, dd (7.0, 1.4)	1.88, dd (7.0, 1.5)	1.87, dd (7.0, 1.6)
4′			6.52, s	6.38, s	6.23, s
6′					6.22, s
8′	2.50, s	2.50, s	2.48, s	2.51, s	2.39, s
8-OAc	2.17, s	2.16, s			2.15, s
3′-OMe	3.83, s	3.83, s	3.83, s	3.92, ^c^ s	3.74, s
5′-OH	6.04, s	6.04, s			
5′-OMe				3.89, ^c^ s	

^a^ Measured at 600 MHz; ^b^ Measured at 700 MHz; ^c^ interchangeable.

**Table 3:** IC_50_ values (µM) of the compounds tested in MDA-MB-231 cells for NF-κB inhibition and inhibition of cell viability. ^a^

Compound	NF-κB inhibition	Cell viability inhibition
**Falconensin O (1)**	15.7 ± 0.7	> 200
**Falconensin A (3)**	53.2 ± 21.4	89.7 ± 9.1
**Falconensin M (4)**	56.5 ± 8.3	> 200
**Falconensin N (5)**	71.0 ± 7.3	> 200
**Falconensin H (6)**	72.0 ± 28.1	> 400
**Falconensin Q (7)**	11.9 ± 2.1	> 200
**Falconensin R (8)**	14.6 ± 1.7	126.8 ± 5.4
**Falconensin S (9)**	20.1 ± 5.6	> 200
**Falconensin I (11)**	19.5 ± 2.5	> 400

^a^Average IC_50_ of at least three individual experiments ± SD tested in the concentration range of 400 µM to 0.78 µM (**3**, **6**, and **11**) and 200 µM to 0.78 µM (**1, 4**, **5**, **7**, **8**, and **9)** in a two-fold serial dilution.

## Supplementary Materials

The following are available online at https://www.mdpi.com/1660-3397/18/4/204/s1, UV, HRMS, 1D and 2D NMR spectra of all the new compounds **1**, **2** and **7**−**9** as well as NF-κB inhibitory potential of the tested compounds and crystal data for compound **5**.
