# Supplementary material for: Azaphilones from the Red Sea Fungus Aspergillus falconensis
Source: Mar Drugs. 2020 Apr 10;18(4):204. doi: 10.3390/md18040204 (PMC7231052; doi:10.3390/md18040204)
Supplement: Supplementary file 1 [file marinedrugs-18-00204-s001.pdf]

Support Information

Azaphilones from the Red Sea Fungus *Aspergillus falconensis*

Dina H. El-Kashef [1,2], Fadia S. Youssef [1,3], Rudolf Hartmann [4], Tim-Oliver Knedel [5], Christoph Janiak [5], Wenhan Lin [6], Irene Reimche [7], Nicole Teusch [7], Zhen Liu [1,*] and Peter Proksch [1,8,*]

1 Institute of Pharmaceutical Biology and Biotechnology, Heinrich-Heine-University Duesseldorf, 40225 Duesseldorf, Germany; dina.elkashef@mu.edu.eg (D.H.E.); fadiayoussef@pharma.asu.edu.eg (F.S.Y.)
2 Department of Pharmacognosy, Faculty of Pharmacy, Minia University, 61519 Minia, Egypt;
3 Department of Pharmacognosy, Faculty of Pharmacy, Ain Shams University, Abbassia, 11566 Cairo, Egypt;
4 Institute of Complex Systems: Strukturbiochemie, Forschungszentrum Jülich GmbH, ICS-6, 52425 Jülich, Germany; r.hartmann@fz-juelich.de (R.H.)
5 Institut für Anorganische Chemie und Strukturchemie, Heinrich-Heine-Universität Düsseldorf, 40225 Düsseldorf, Germany; tim-oliver.knedel@hhu.de (T.O.K.); janiak@uni-duesseldorf.de (C.J.)
6 State Key Laboratory of Natural and Biomimetic Drugs, Peking University, Beijing 100191, China; whlin@bjmu.edu.cn (W.L.)
7 Department of Biomedical Sciences, Institute of Health Research and Education, University of Osnabrück, Germany; irene.reimche@uni-osnabrueck.de (I.R.); nicole.teusch@uni-osnabrueck.de (N.T.)
8 Hubei Key Laboratory of Natural Products Research and Development, College of Biological and Pharmaceutical Sciences, China Three Gorges University, Yichang 443002, People's Republic of China;
* Correspondence: zhenfeizi0@sina.com (Z.L.); proksch@uni-duesseldorf.de (P.P.); Tel.: +49-211-81-14163

Contents

Figure S1. UV spectrum of compound **1**. 4
Figure S2. HRESIMS of compound **1**. 4
Figure S3. ^{1}H NMR (600 MHz, $CDCl_3$) spectrum of compound **1**. 5
Figure S4. COSY (600 MHz, $CDCl_3$) spectrum of compound **1** 6
Figure S5. HSQC (600 MHz/150 MHz, $CDCl_3$) spectrum of compound **1**. 7
Figure S6. HMBC (600 MHz/150 MHz, $CDCl_3$) spectrum of compound **1**. 8
Figure S7. ROESY (600 MHz, $CDCl_3$) spectrum of compound **1**. 9
Figure S8. UV spectrum of compound **2**. 10
Figure S9. HRESIMS of compound **2**. 10
Figure S10. ^{1}H NMR (600 MHz, $CDCl_3$) spectrum of compound **2**. 11
Figure S11. COSY (600 MHz, $CDCl_3$) spectrum of compound **2** 12
Figure S12. HSQC (600 MHz/150 MHz, $CDCl_3$) spectrum of compound **2**. 13
Figure S13. HMBC (600 MHz/150 MHz, $CDCl_3$) spectrum of compound **2**. 14
Figure S14. ROESY (600 MHz, $CDCl_3$) spectrum of compound **2**. 15
Figure S15. UV spectrum of compound **7**. 16
Figure S16. HRESIMS of compound **7**. 16
Figure S17. ^{1}H NMR (700 MHz, $CDCl_3$) spectrum of compound **7**. 17
Figure S18. ^{13}C NMR (175 MHz, $CDCl_3$) spectrum of compound **7**. 18
Figure S19. COSY (700 MHz, $CDCl_3$) spectrum of compound **7** 19
Figure S20. HSQC (700 MHz/175 MHz, $CDCl_3$) spectrum of compound **7**. 20
Figure S21. HMBC (700 MHz/175 MHz, $CDCl_3$) spectrum of compound **7**. 21
Figure S22. ROESY (700 MHz/175 MHz, $CDCl_3$) spectrum of compound **7**. 22
Figure S23. UV spectrum of compound **8**. 23
Figure S24. HRESIMS of compound **8**. 23
Figure S25. ^{1}H NMR (600 MHz, $CDCl_3$) spectrum of compound **8**. 24
Figure S26. COSY (600 MHz, $CDCl_3$) spectrum of compound **8** 25
Figure S27. HSQC (600 MHz/150 MHz, $CDCl_3$) spectrum of compound **8**. 26
Figure S28. HMBC (600 MHz/150 MHz, $CDCl_3$) spectrum of compound **8**. 27
Figure S29. ROESY (600 MHz, $CDCl_3$) spectrum of compound **8**. 28
Figure S30. UV spectrum of compound **9** 29
Figure S31. HRESIMS of compound **9**. 29
Figure S32. ^{1}H NMR (700 MHz, $CDCl_3$) spectrum of compound **9**. 30
Figure S33. ^{13}C NMR (175 MHz, $CDCl_3$) spectrum of compound **9**. 31
Figure S34. COSY (700 MHz, $CDCl_3$) spectrum of compound **9** 32

Figure S35. HSQC (700 MHz/175 MHz, $CDCl_3$) spectrum of compound **9**. 33

Figure S36. HMBC (700 MHz/175 MHz, $CDCl_3$) spectrum of compound **9**. 34

Figure S37. ROESY (700 MHz, $CDCl_3$) spectrum of compound **9**. 35

Figure S38. Section of the packing diagram over an extended unit cell of compound **5** (50% thermal elipsoids) determined by single-crystal x-ray diffraction. Hydrogen bonds are shown in dashed yellow lines. 36

Figure S39. NFκB inhibitory potential of the compounds **1**, **3,** **4**, **5**, **6**, **7**, **8**, **9** and **11**........ 37

Figure S40. Potency in NFκB inhibition versus cytotoxicity. 38

Table S1: Crystal data for compound **5**. 39

Table S2. Fractional atomic coordinates and isotropic or equivalent isotropic displacement parameters ($Å^2$) for compound **5**........ 40

Table S3. Atomic displacement parameters ($Å^2$) for compound **5**. 42

Table S4. Geometric parameters (Å, º) for compound **5**. 43

Table S5. Hydrogen-bond geometry (Å, º) for compound **5**. 45

Figure S1. UV spectrum of compound **1**.

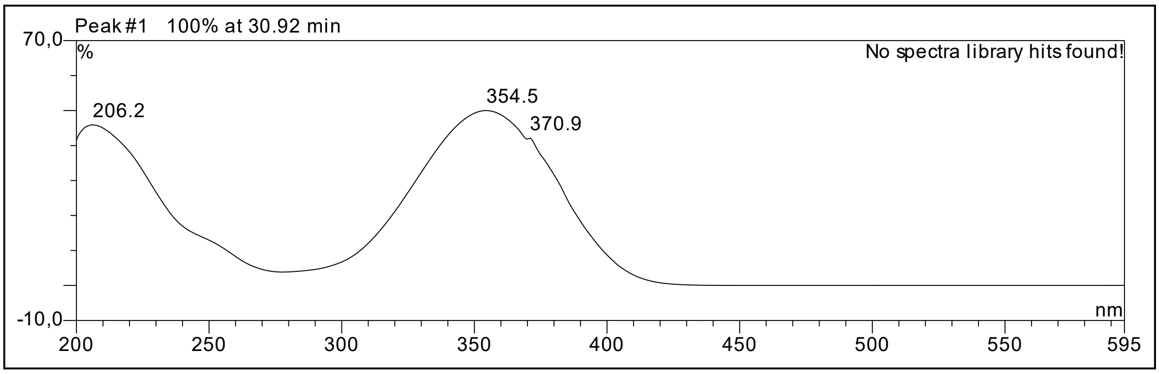

Figure S2. HRESIMS of compound **1**.

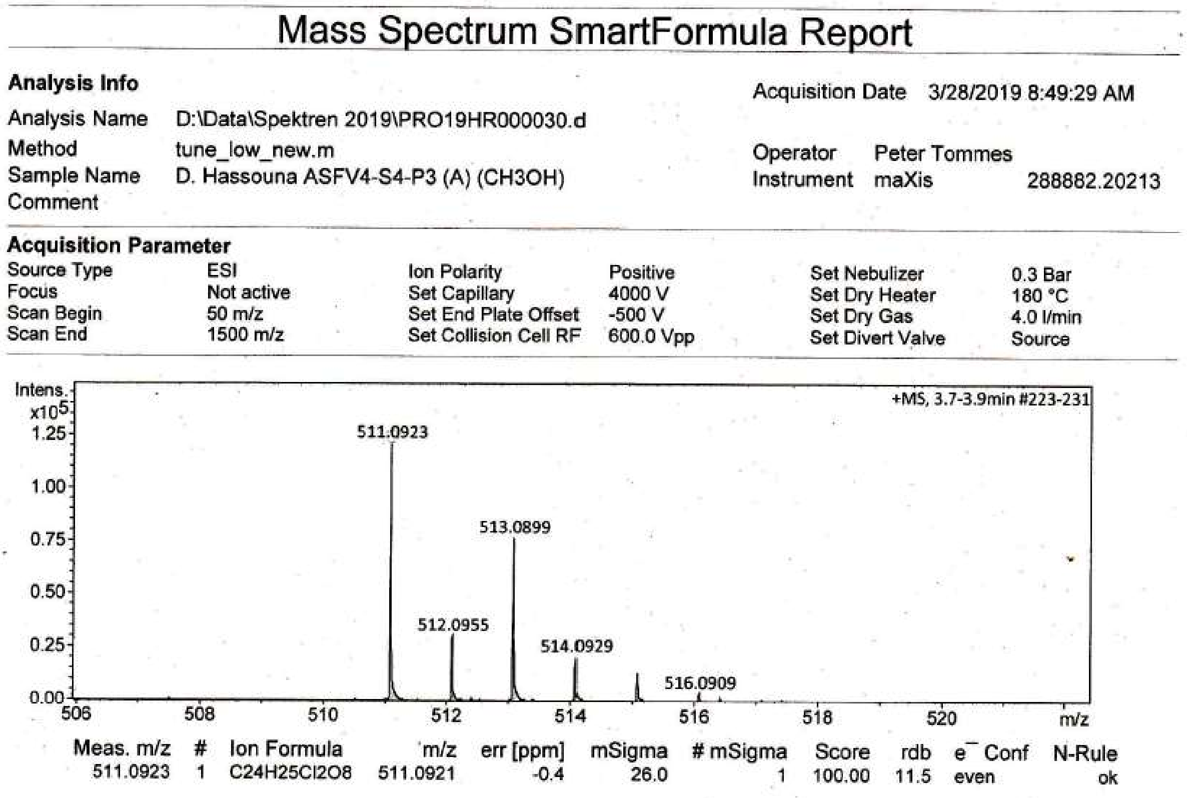
Mass Spectrum SmartFormula Report

Analysis Info

Analysis Name D:\Data\Spektren 2019\PRO19HR000030.d
Method tune_low_new.m
Sample Name D. Hassouna ASFV4-S4-P3 (A) (CH3OH)
Comment

Acquisition Date 3/28/2019 8:49:29 AM
Operator Peter Tommes
Instrument maXis 288882.20213

Acquisition Parameter

Source Type	ESI	Ion Polarity	Positive	Set Nebulizer	0.3 Bar
Focus	Not active	Set Capillary	4000 V	Set Dry Heater	180 °C
Scan Begin	50 m/z	Set End Plate Offset	-500 V	Set Dry Gas	4.0 l/min
Scan End	1500 m/z	Set Collision Cell RF	600.0 Vpp	Set Divert Valve	Source

Meas. m/z	#	Ion Formula	m/z	err [ppm]	mSigma	# mSigma	Score	rdb	e⁻ Conf	N-Rule
511.0923	1	C24H25Cl2O8	511.0921	-0.4	26.0	1	100.00	11.5	even	ok

Figure S3. ^{1}H NMR (600 MHz, $CDCl_3$) spectrum of compound **1**.

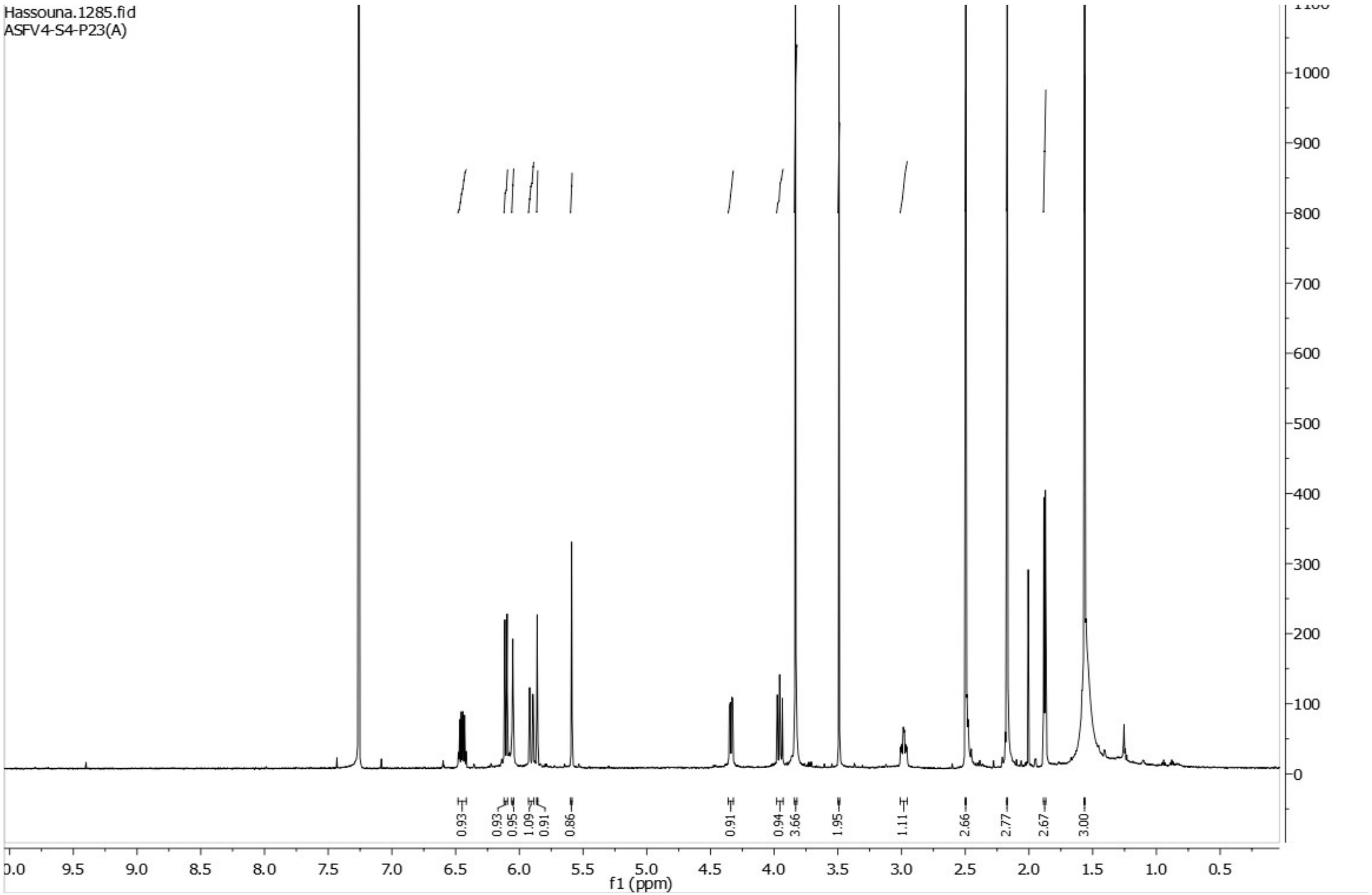

Figure S4. COSY (600 MHz, $CDCl_3$) spectrum of compound **1**.

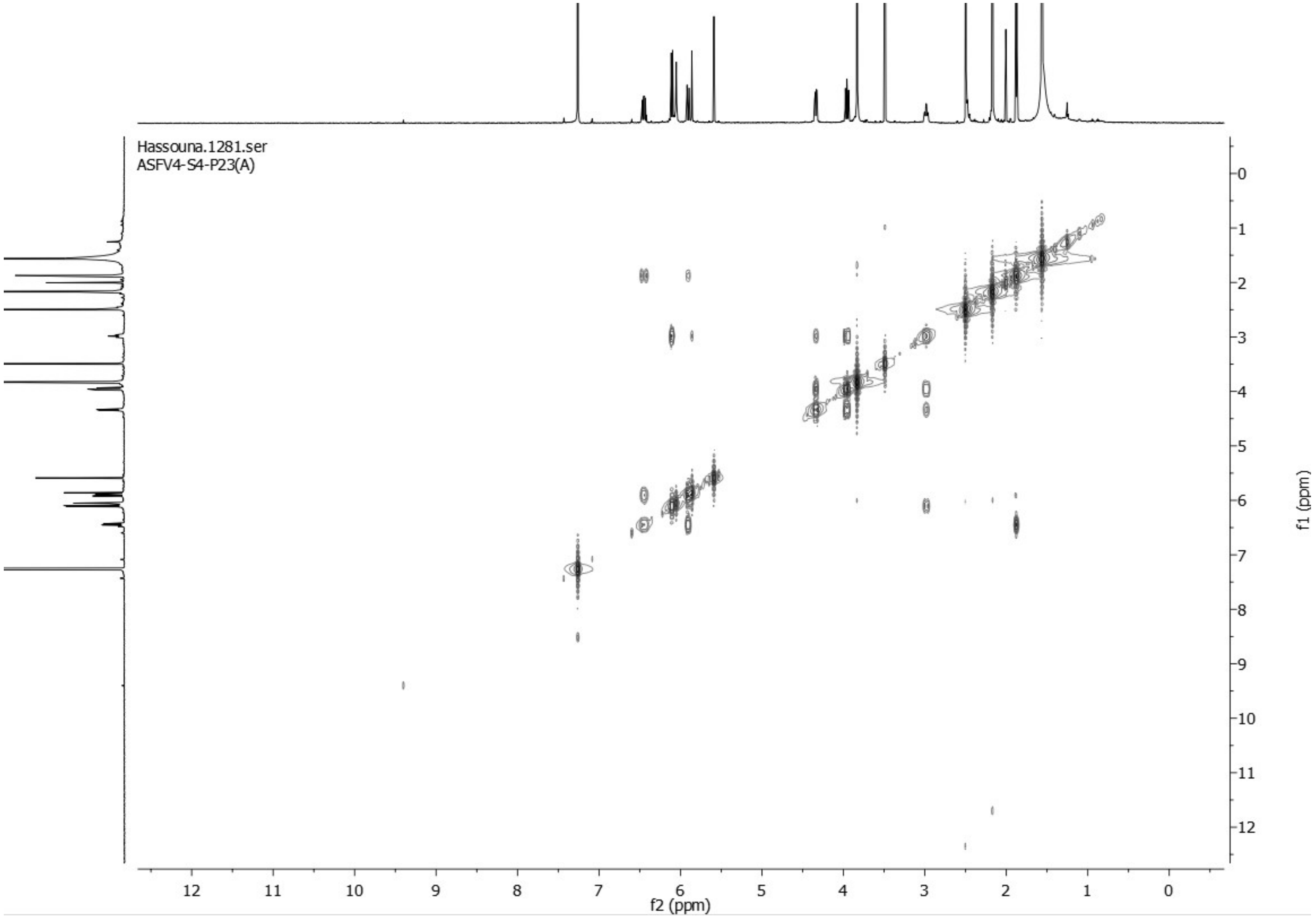

Figure S5. HSQC (600 MHz/150 MHz, $CDCl_3$) spectrum of compound **1**.

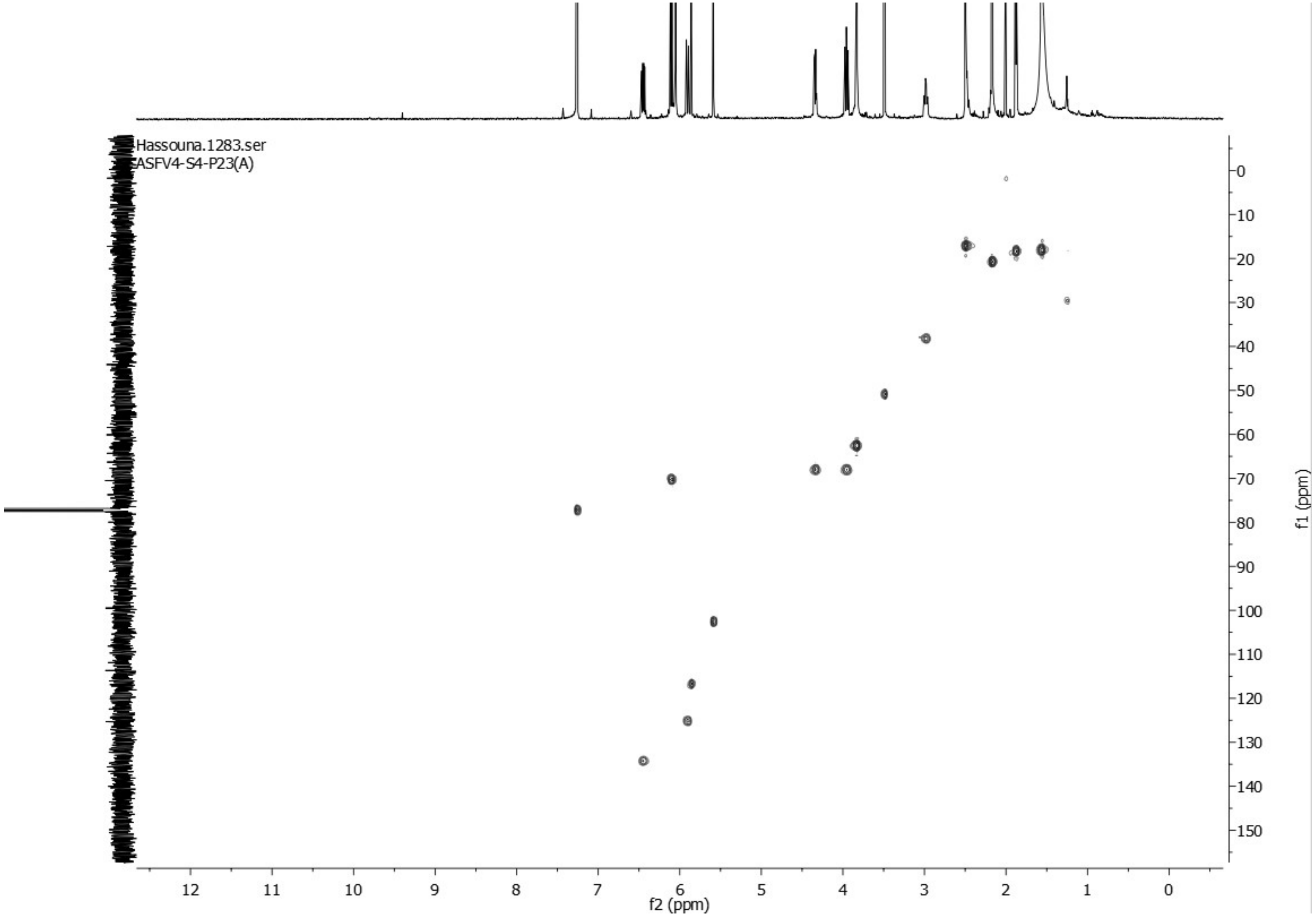

Figure S6. HMBC (600 MHz/150 MHz, $CDCl_3$) spectrum of compound **1**.

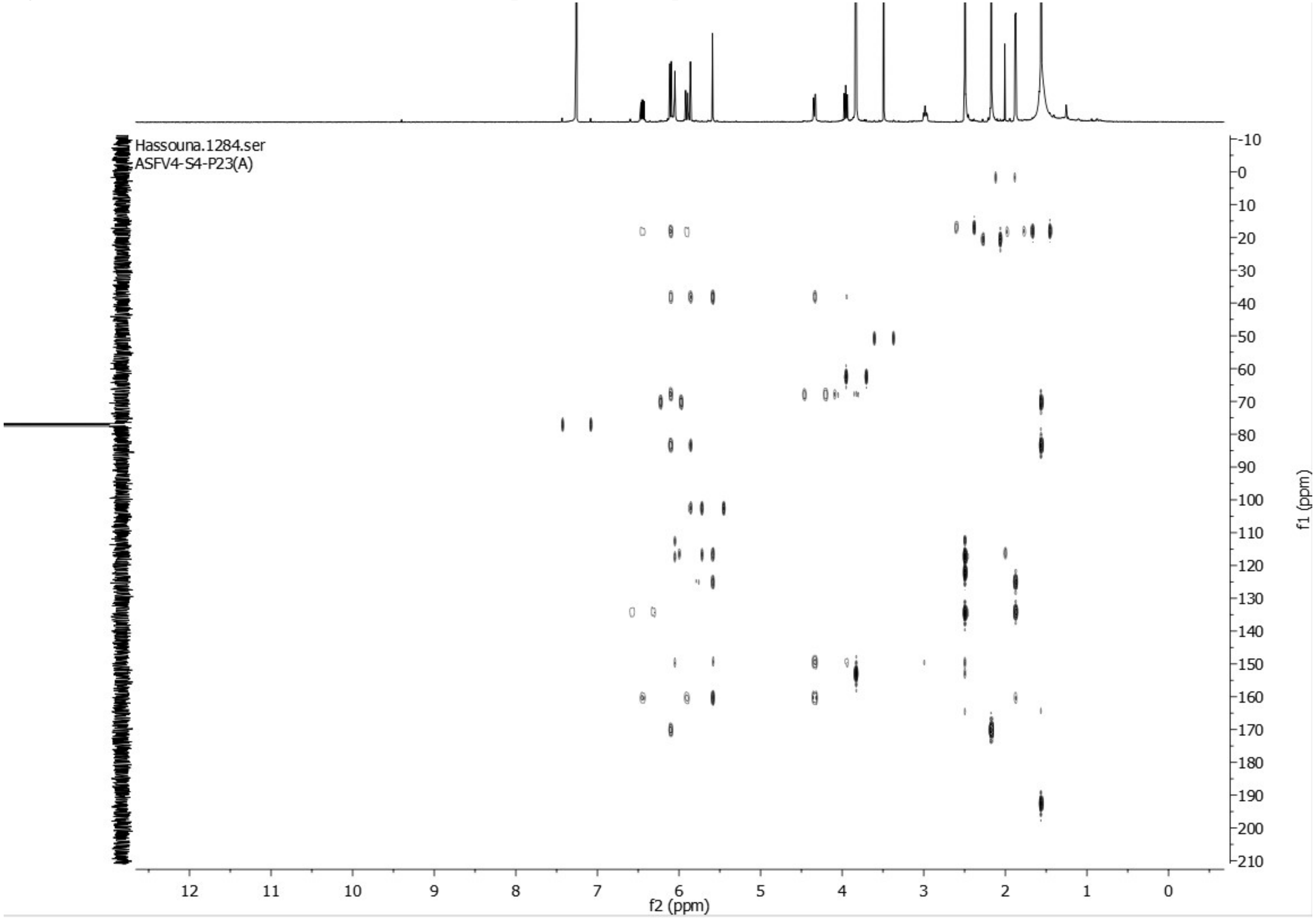

Figure S7. ROESY (600 MHz, $CDCl_3$) spectrum of compound **1**.

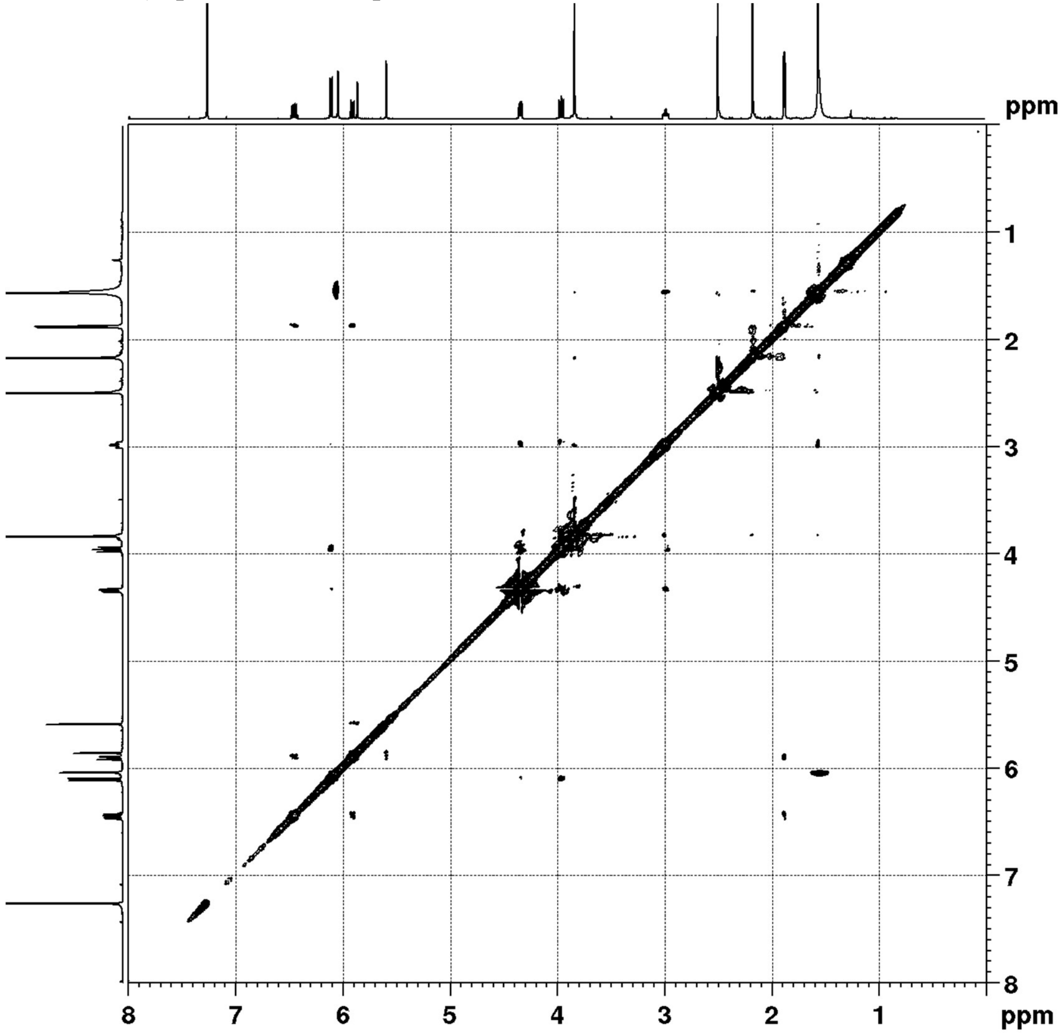

Figure S8. UV spectrum of compound **2**.

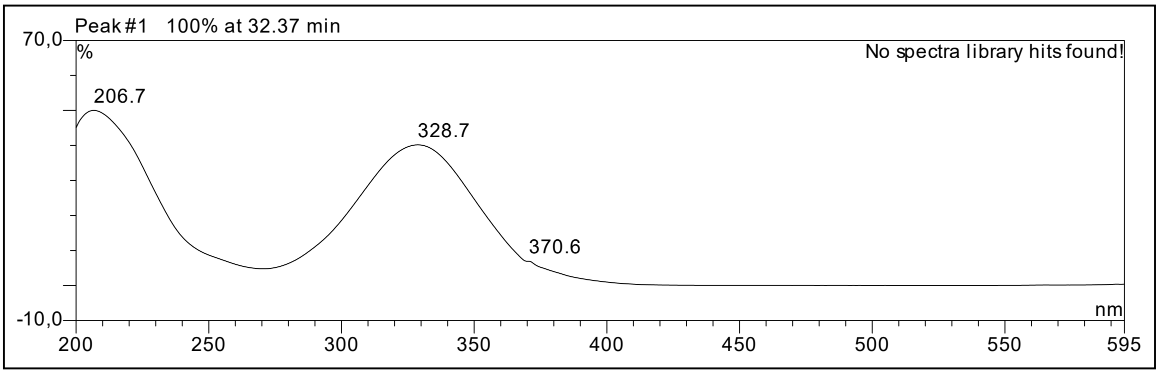

Figure S9. HRESIMS of compound **2**.

Mass Spectrum SmartFormula Report

Analysis Info

Analysis Name	D:\Data\Spektren 2019\PRO19HR000031.d	Acquisition Date	3/28/2019 9:04:42 AM
Method	tune_low_new.m	Operator	Peter Tommes
Sample Name	D. Hassouna ASFV4-S4-P3 (C) (CH3OH)	Instrument	maXis 288882.20213
Comment			

Acquisition Parameter

Source Type	ESI	Ion Polarity	Positive	Set Nebulizer	0.3 Bar
Focus	Not active	Set Capillary	4000 V	Set Dry Heater	180 °C
Scan Begin	50 m/z	Set End Plate Offset	-500 V	Set Dry Gas	4.0 l/min
Scan End	1500 m/z	Set Collision Cell RF	600.0 Vpp	Set Divert Valve	Source

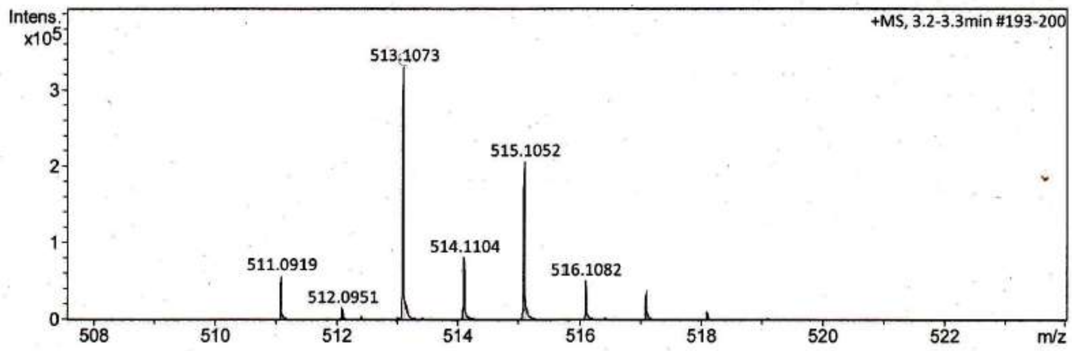

Meas. m/z	#	Ion Formula	m/z	err [ppm]	mSigma	# mSigma	Score	rdb	e⁻ Conf	N-Rule
513.1073	1	C21H19Cl2N10O2	513.1064	-1.7	27.3	1	93.28	16.5	even	ok
	2	C24H27Cl2O8	513.1077	0.9	32.8	2	100.00	10.5	even	ok

Figure S10. ^{1}H NMR (600 MHz, $CDCl_3$) spectrum of compound **2**.

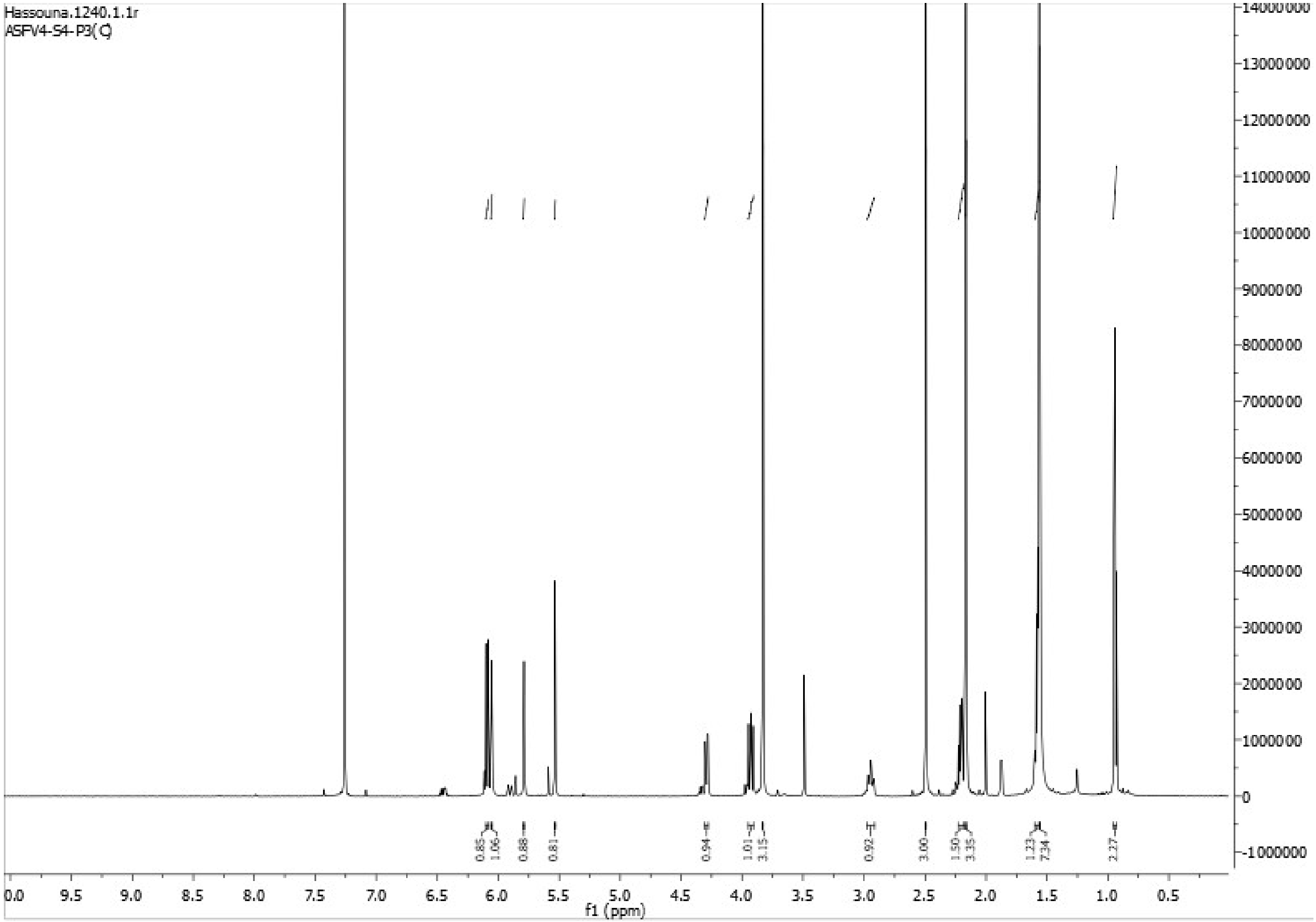

Figure S11. COSY (600 MHz, $CDCl_3$) spectrum of compound **2**.

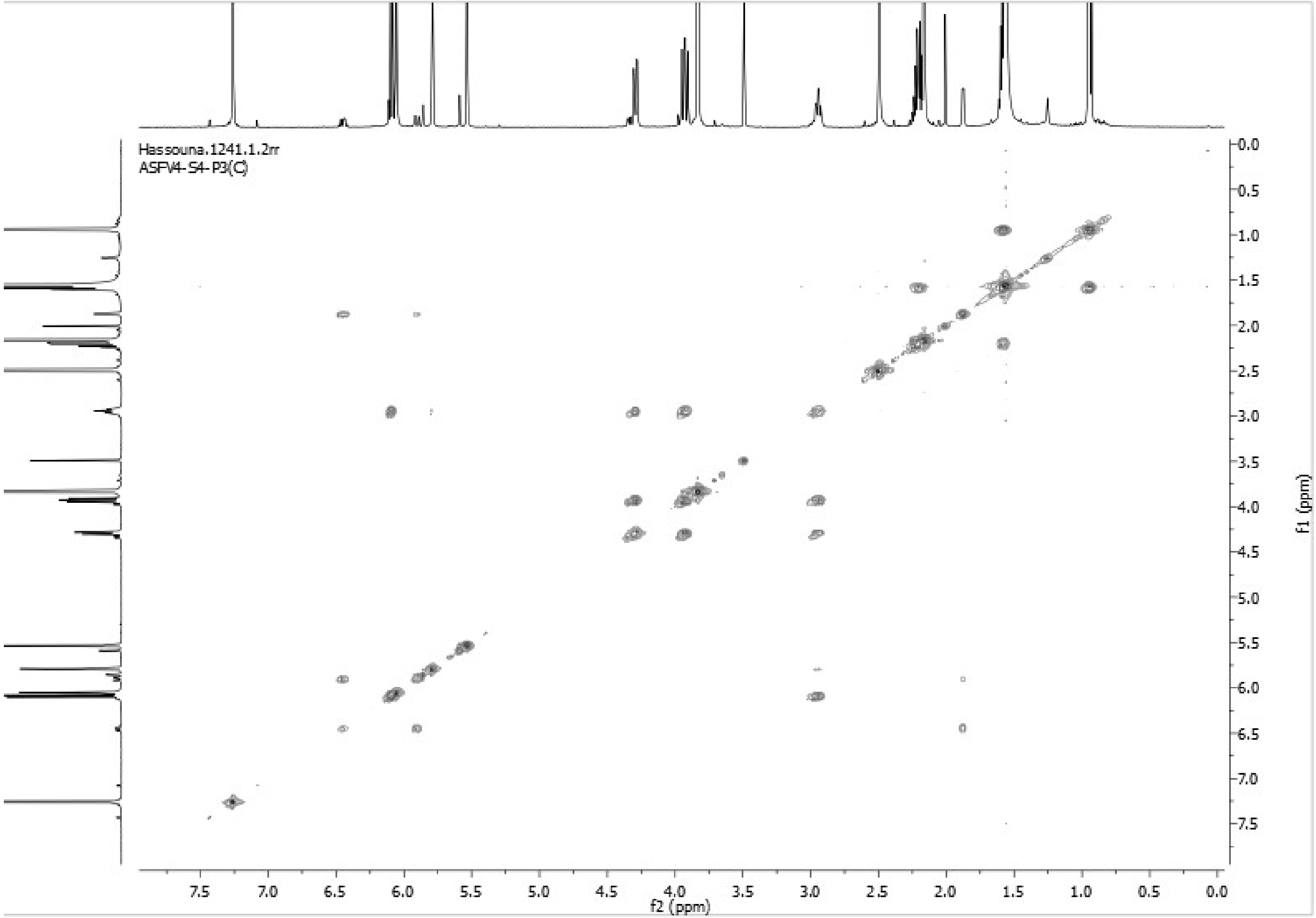

Figure S12. HSQC (600 MHz/150 MHz, $CDCl_3$) spectrum of compound **2**.

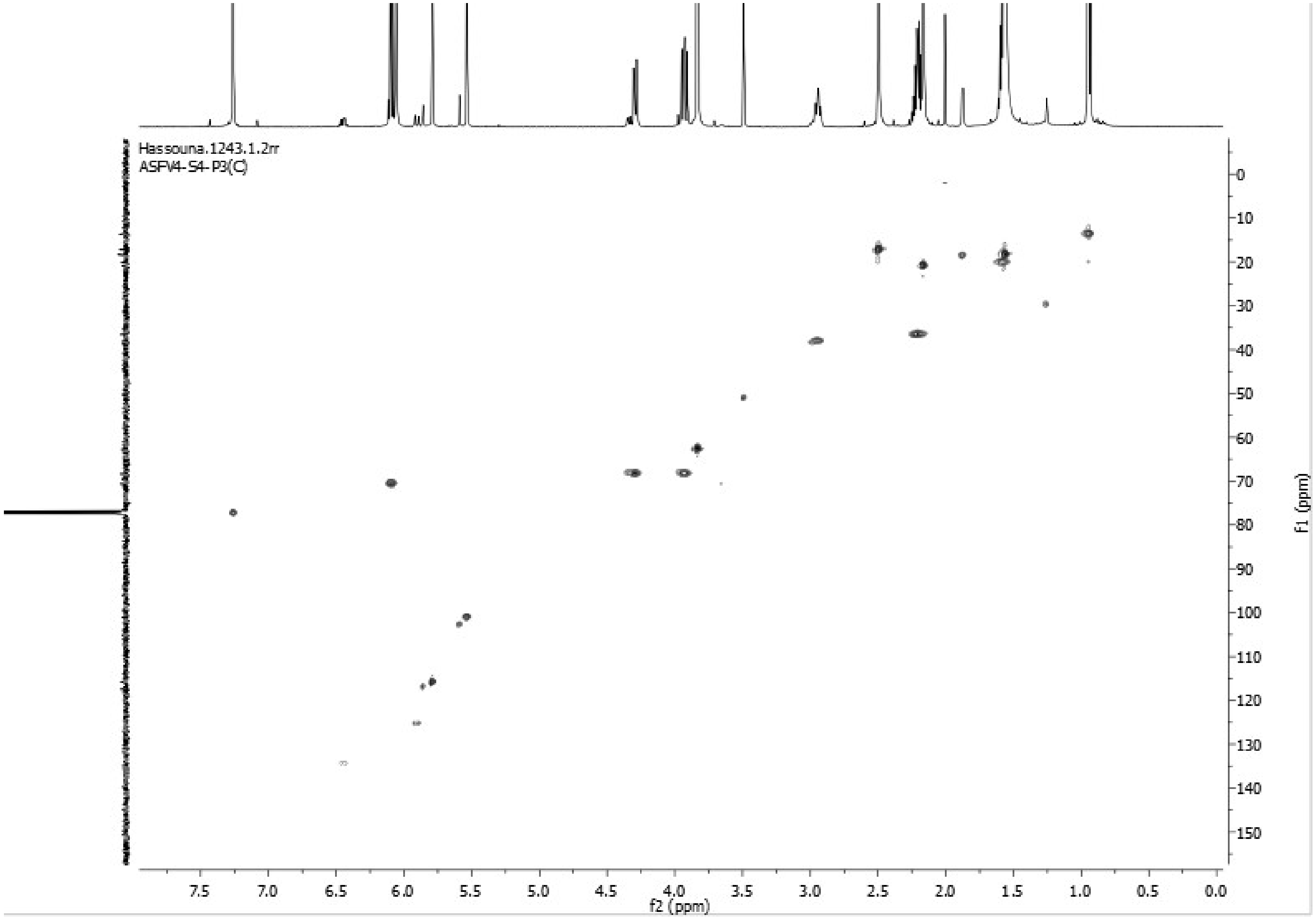

Figure S13. HMBC (600 MHz/150 MHz, $CDCl_3$) spectrum of compound **2**.

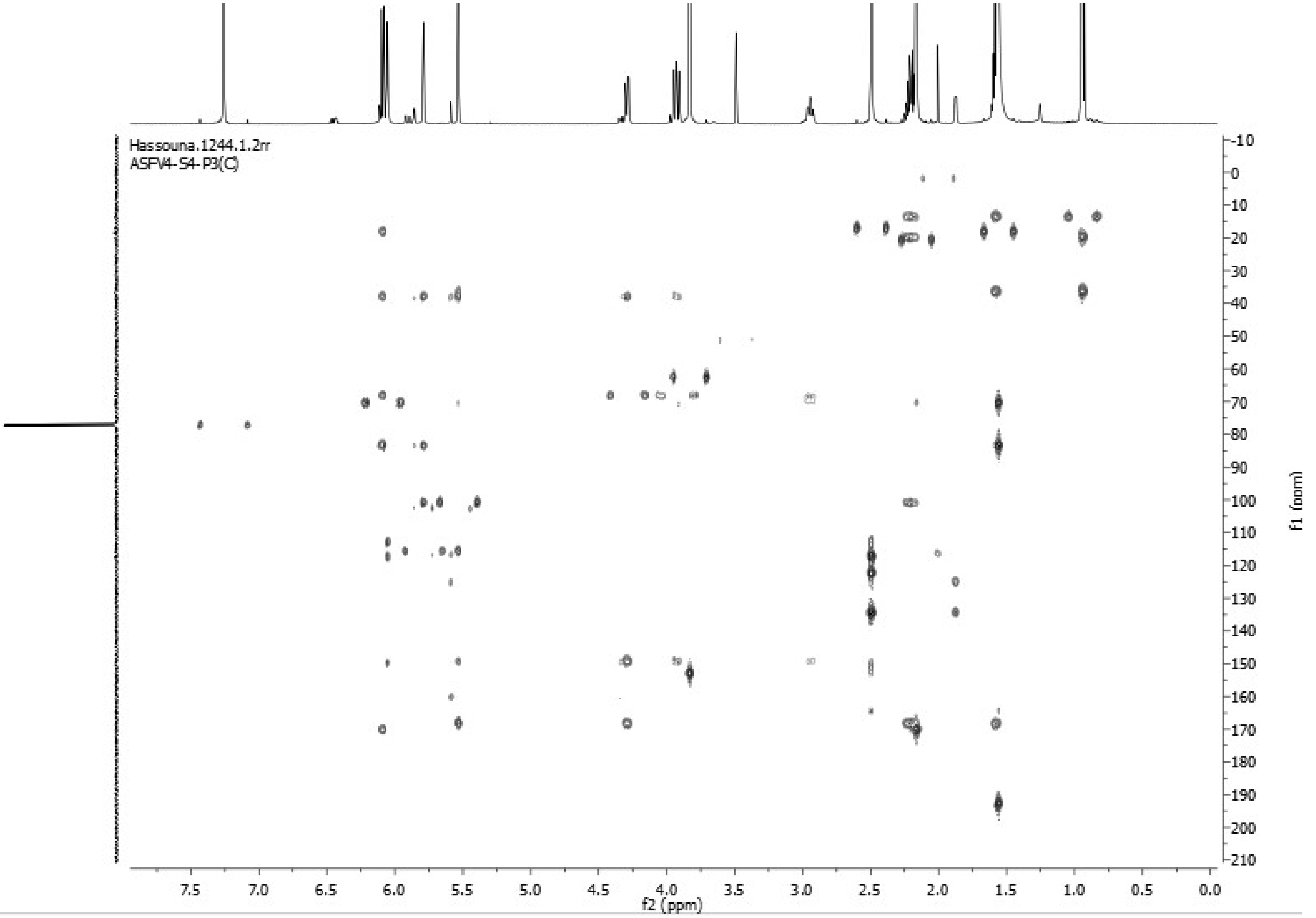

Figure S14. ROESY (600 MHz, $CDCl_3$) spectrum of compound **2**.

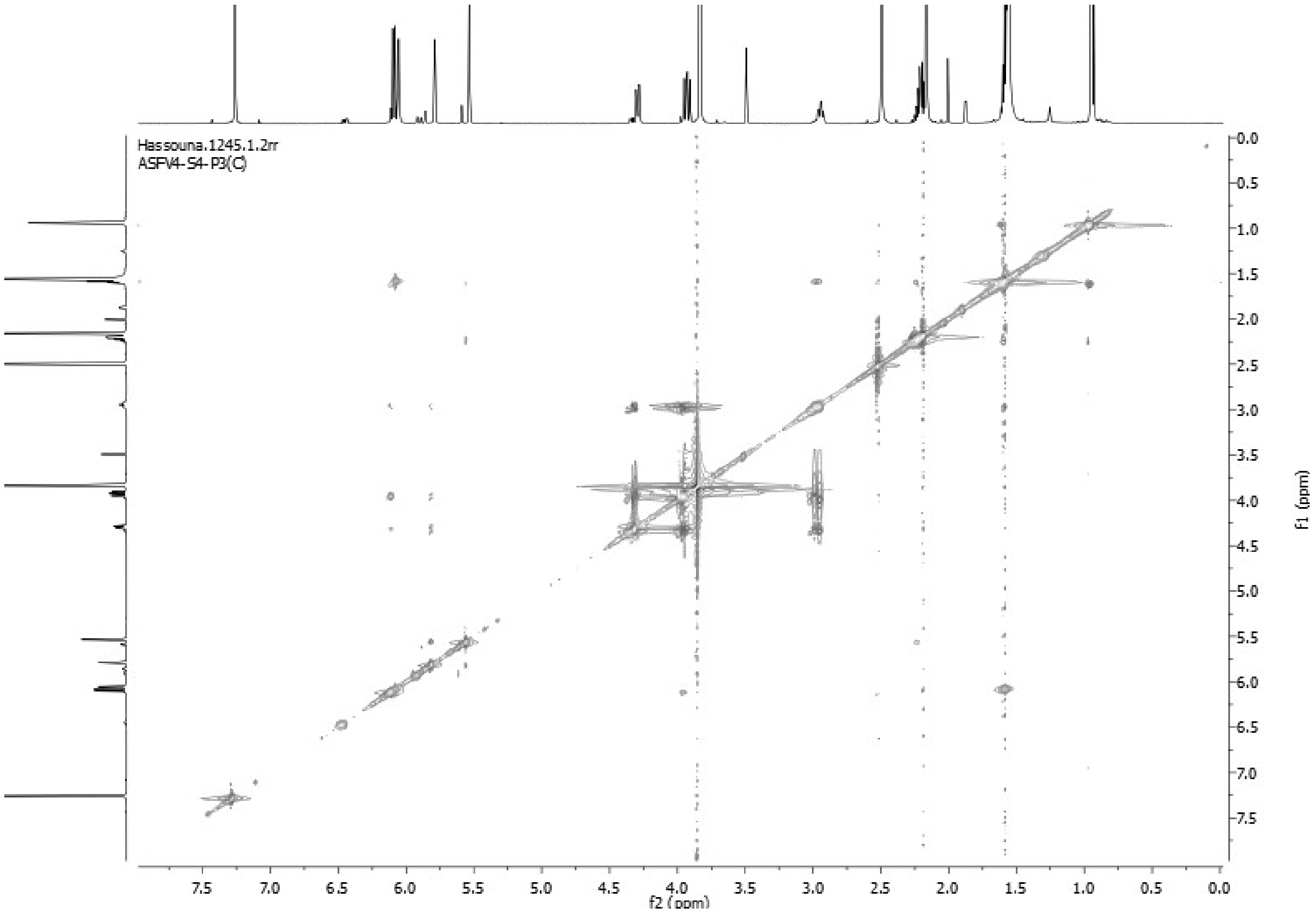

Figure S15. UV spectrum of compound **7**.

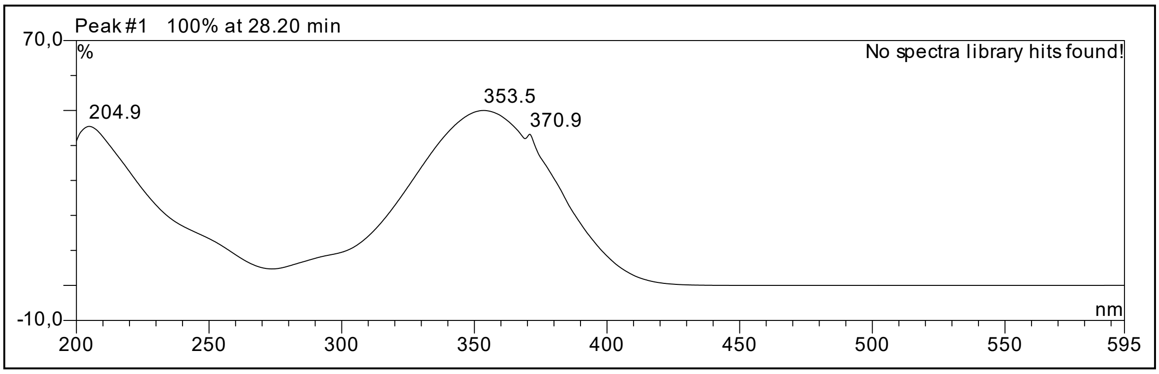

Figure S16. HRESIMS of compound **7**.

Mass Spectrum SmartFormula Report

Analysis Info

Analysis Name	D:\Data\Spektren 2019\PRO19HR000028.d	Acquisition Date	3/27/2019 3:52:18 PM
Method	tune_low_new.m	Operator	Peter Tommes
Sample Name	D. Hassouna ASFBr-V5-P8 (CH3OH)	Instrument	maXis 288882.20213
Comment			

Acquisition Parameter

Source Type	ESI	Ion Polarity	Positive	Set Nebulizer	0.3 Bar
Focus	Not active	Set Capillary	4000 V	Set Dry Heater	180 °C
Scan Begin	50 m/z	Set End Plate Offset	-500 V	Set Dry Gas	4.0 l/min
Scan End	1500 m/z	Set Collision Cell RF	600.0 Vpp	Set Divert Valve	Source

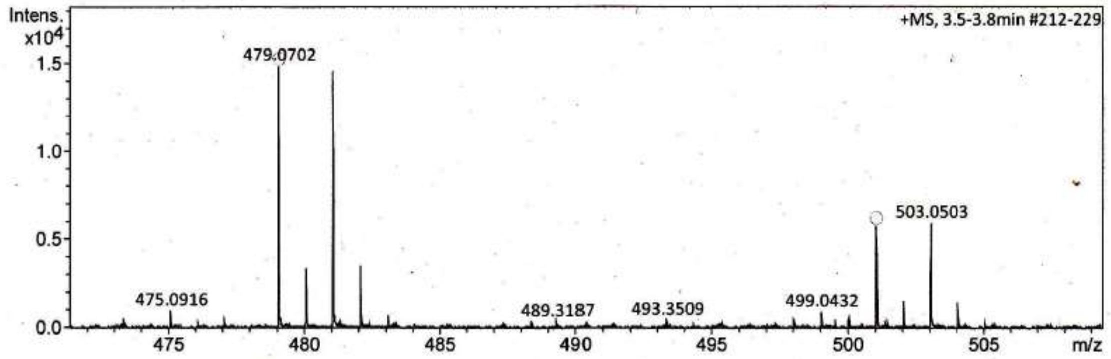

Meas. m/z	#	Ion Formula	m/z	err [ppm]	mSigma	# mSigma	Score	rdb	e⁻ Conf	N-Rule
479.0702	1	C22H24BrO7	479.0700	-0.5	11.9	1	100.00	10.5	even	ok
501.0519	1	C22H23BrNaO7	501.0519	0.1	11.1	1	100.00	10.5	even	ok

Figure S17. ^{1}H NMR (700 MHz, $CDCl_3$) spectrum of compound **7**.

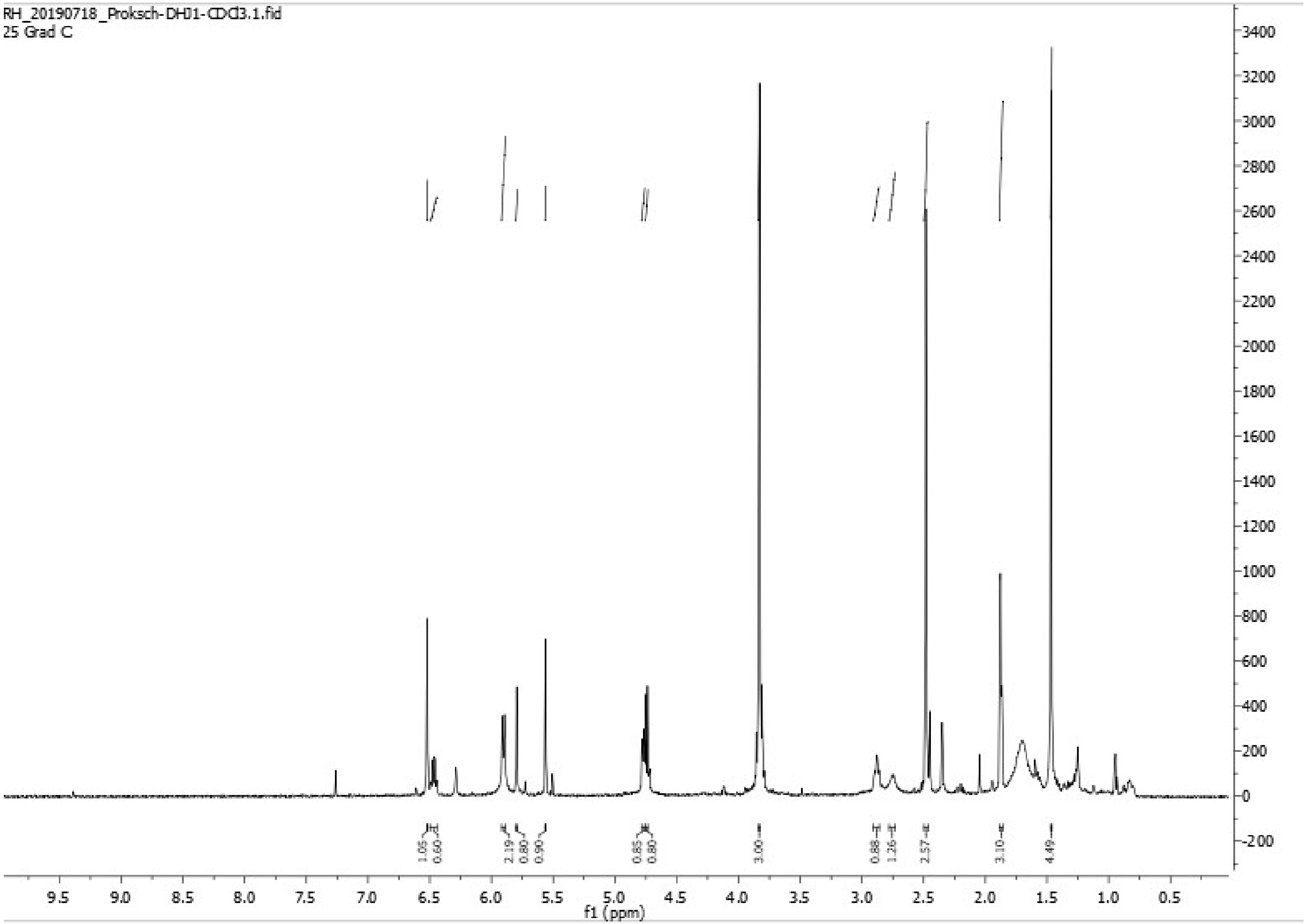

Figure S18. ^{13}C NMR (175 MHz, $CDCl_3$) spectrum of compound **7**.

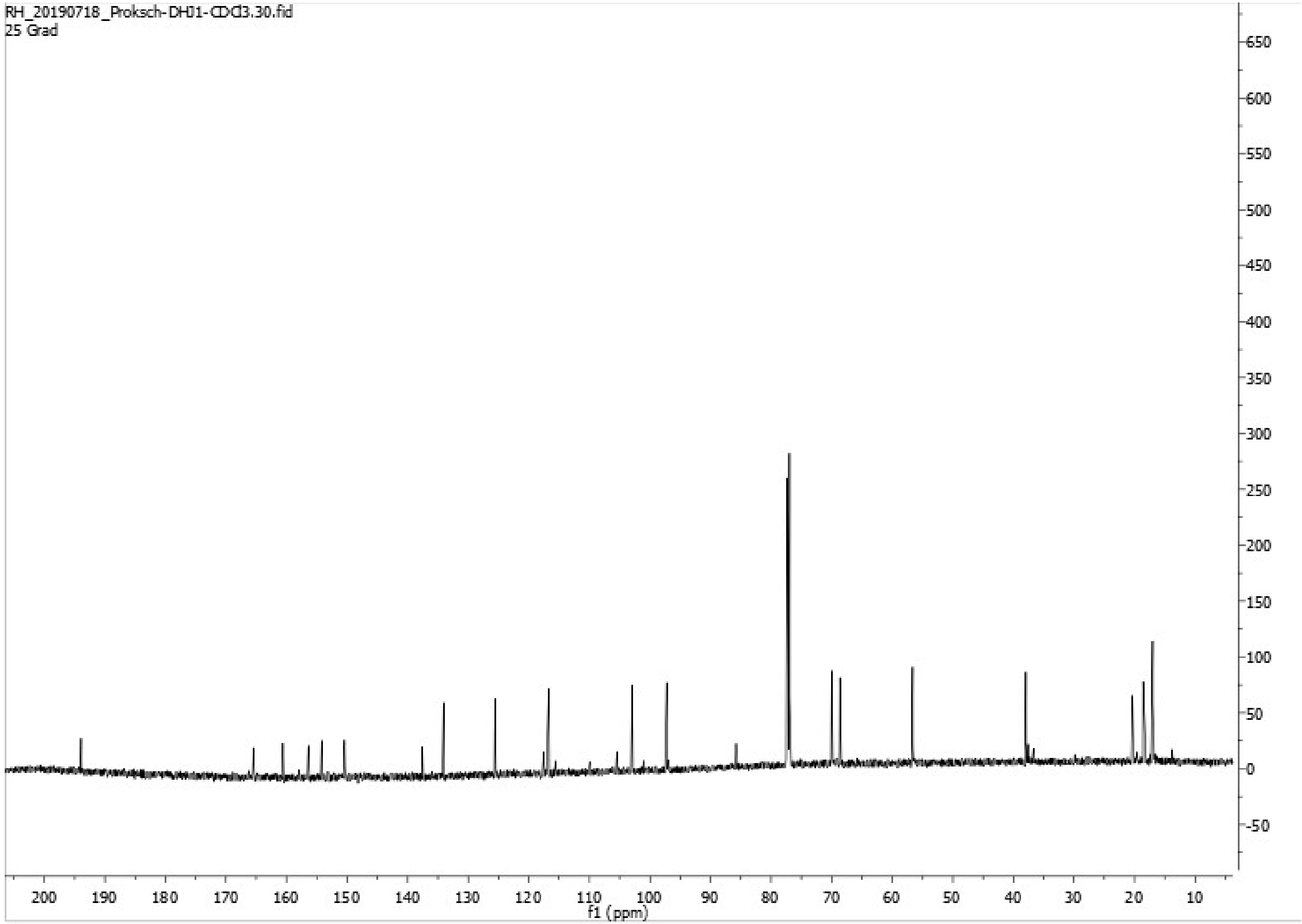

Figure S19. COSY (700 MHz, $CDCl_3$) spectrum of compound **7**.

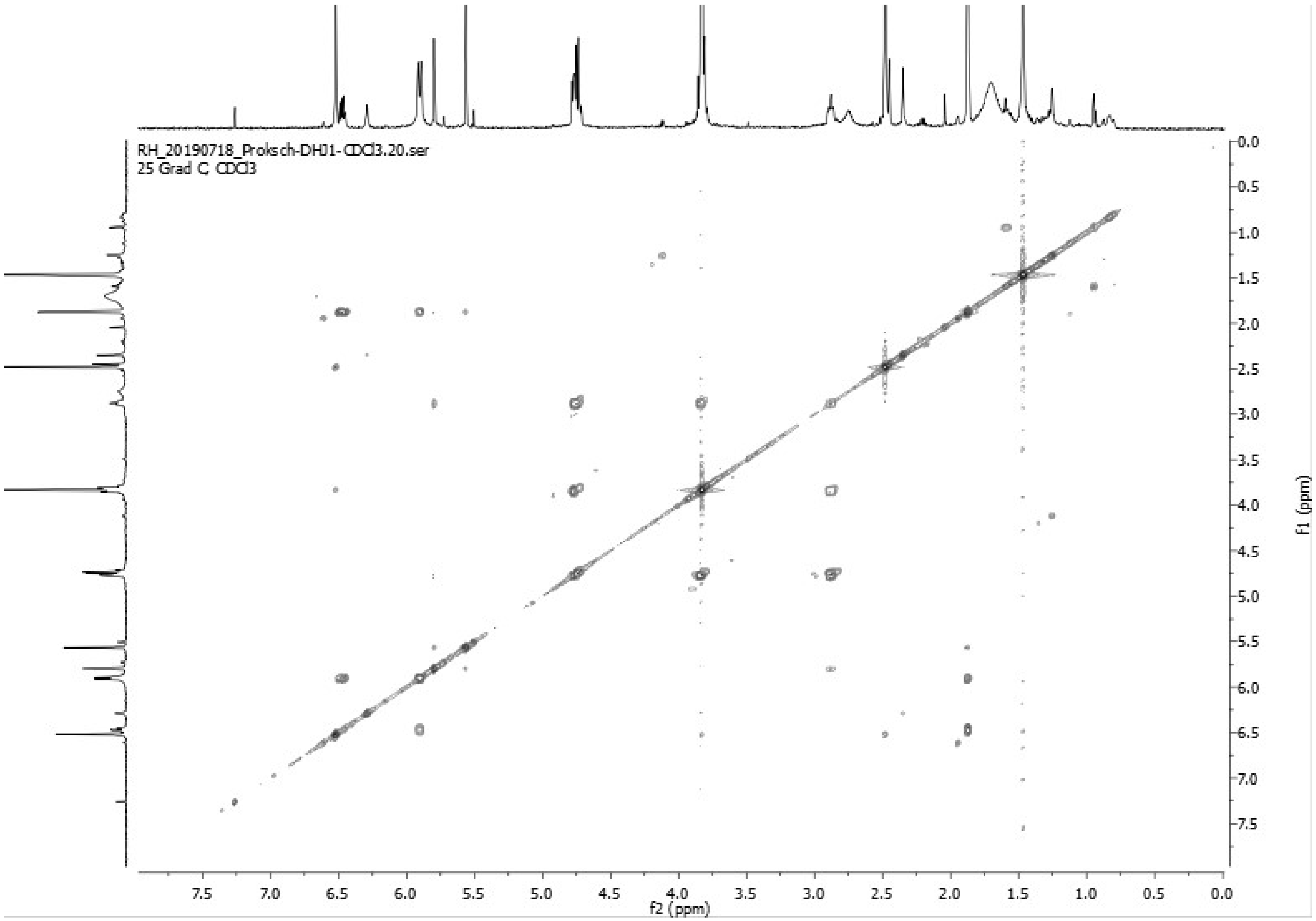

Figure S20. HSQC (700 MHz/175 MHz, $CDCl_3$) spectrum of compound **7**.

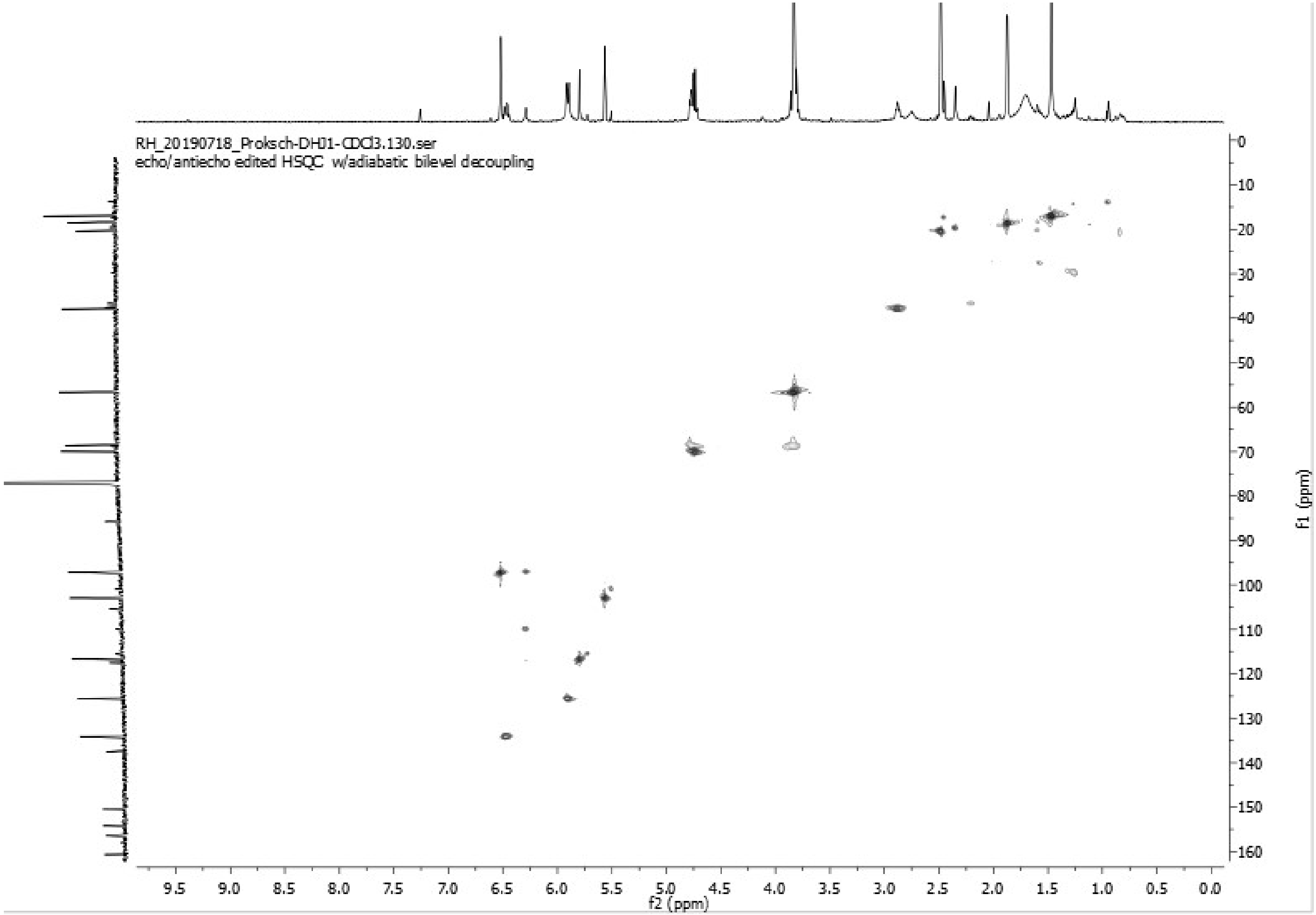

Figure S21. HMBC (700 MHz/175 MHz, $CDCl_3$) spectrum of compound **7**.

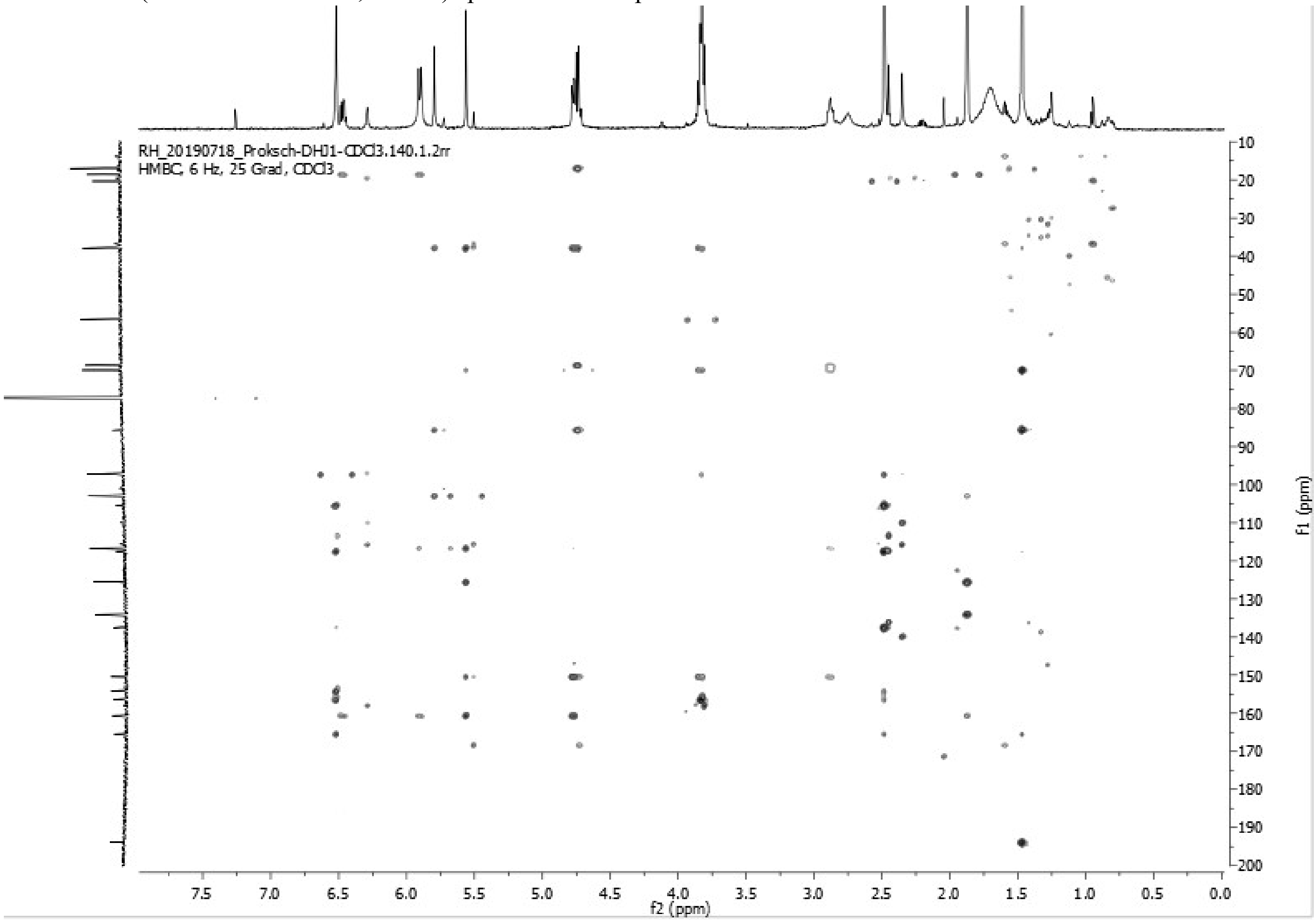

Figure S22. ROESY (700 MHz/175 MHz, $CDCl_3$) spectrum of compound **7**.

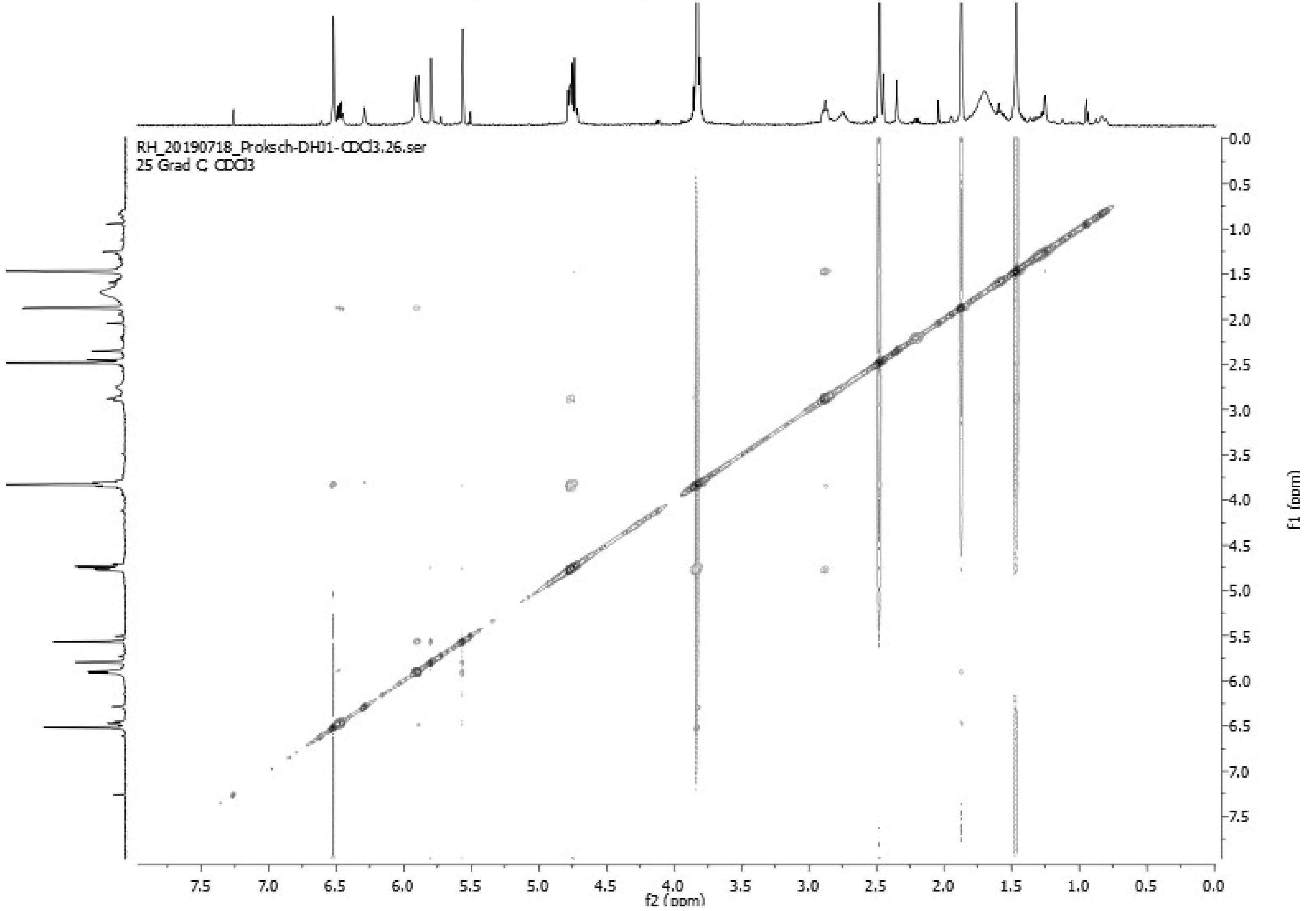

Figure S23. UV spectrum of compound **8**.

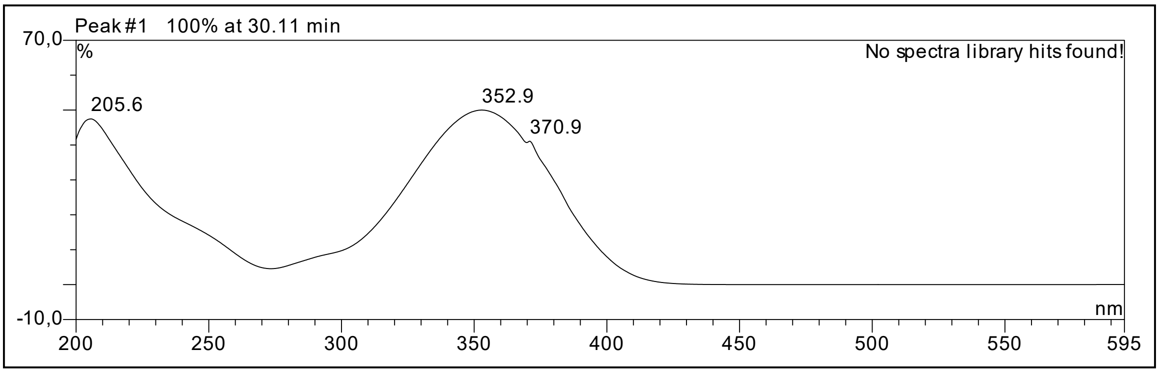

Figure S24. HRESIMS of compound **8**.

Mass Spectrum SmartFormula Report

Analysis Info

Analysis Name	D:\Data\Spektren 2019\PRO19HR000198.d	Acquisition Date	8/30/2019 12:45:23 PM
Method	tune_low_new.m	Operator	Peter Tommes
Sample Name	Hassouna ASFBr-V5-P10 II (CH3OH)	Instrument	maXis 288882.20213
Comment	25 ul in 1 ml		

Acquisition Parameter

Source Type	ESI	Ion Polarity	Positive	Set Nebulizer	0.3 Bar
Focus	Not active	Set Capillary	4000 V	Set Dry Heater	180 °C
Scan Begin	50 m/z	Set End Plate Offset	-500 V	Set Dry Gas	4.0 l/min
Scan End	1500 m/z	Set Collision Cell RF	600.0 Vpp	Set Divert Valve	Source

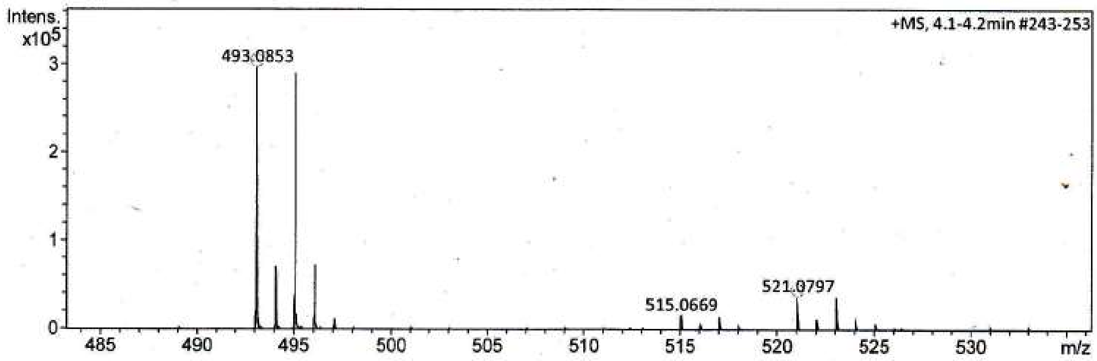

Meas. m/z	#	Ion Formula	m/z	err [ppm]	mSigma	# mSigma	Score	rdb	e⁻ Conf	N-Rule
256.9806	1	C10H10BrO3	256.9808	0.6	22.8	1	100.00	5.5	even	ok
493.0853	1	C20H18BrN10O	493.0843	-2.0	12.1	1	74.41	16.5	even	ok
	2	C23H26BrO7	493.0856	0.8	12.7	2	100.00	10.5	even	ok
521.0797	1	C24H26BrO8	521.0806	1.6	48.1	1	98.38	11.5	even	ok
	2	C21H18BrN10O2	521.0792	-1.0	52.4	2	100.00	17.5	even	ok

Figure S25. ^{1}H NMR (600 MHz, $CDCl_3$) spectrum of compound **8**.

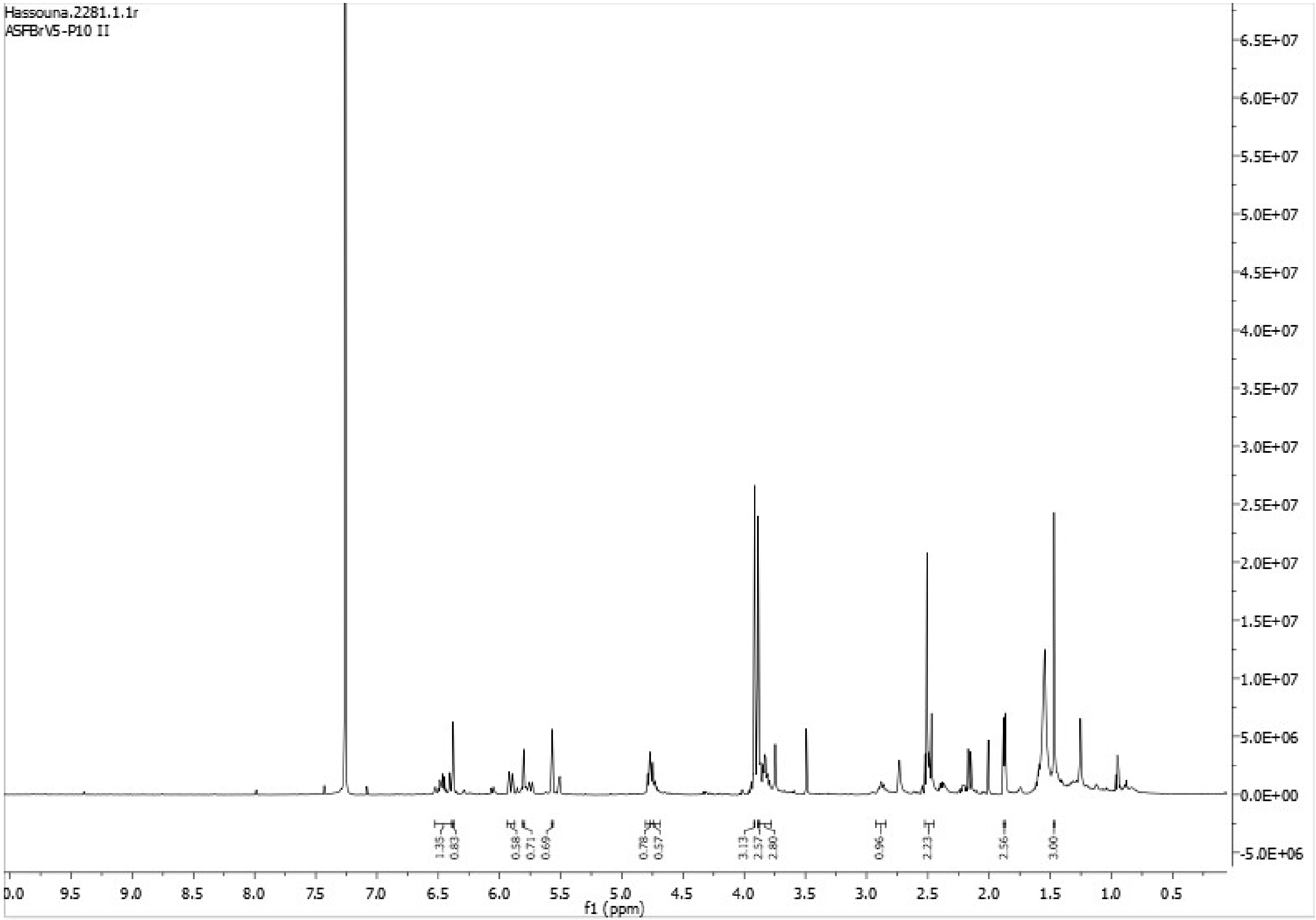

Figure S26. COSY (600 MHz, $CDCl_3$) spectrum of compound **8**.

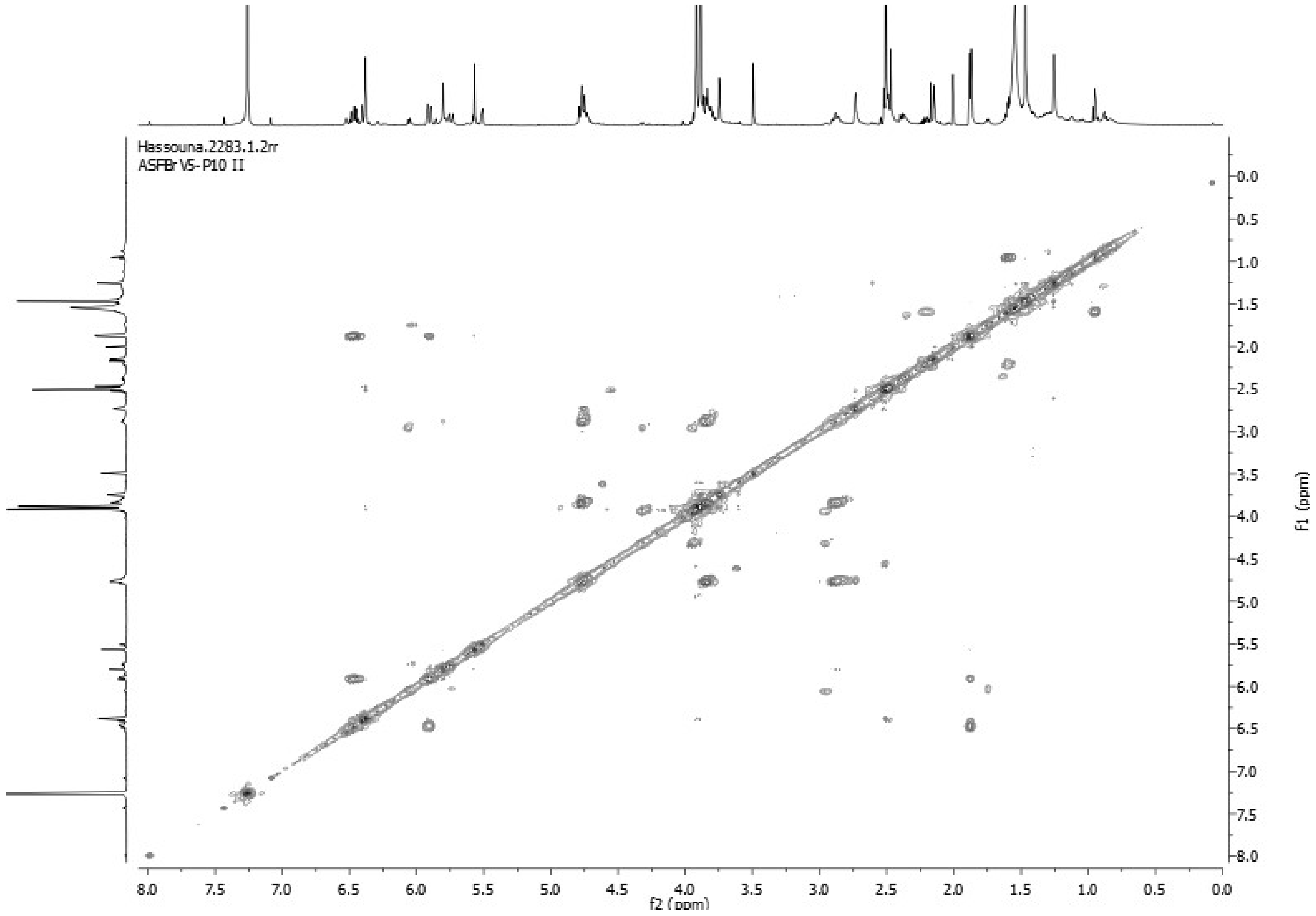

Figure S27. HSQC (600 MHz/150 MHz, $CDCl_3$) spectrum of compound **8**.

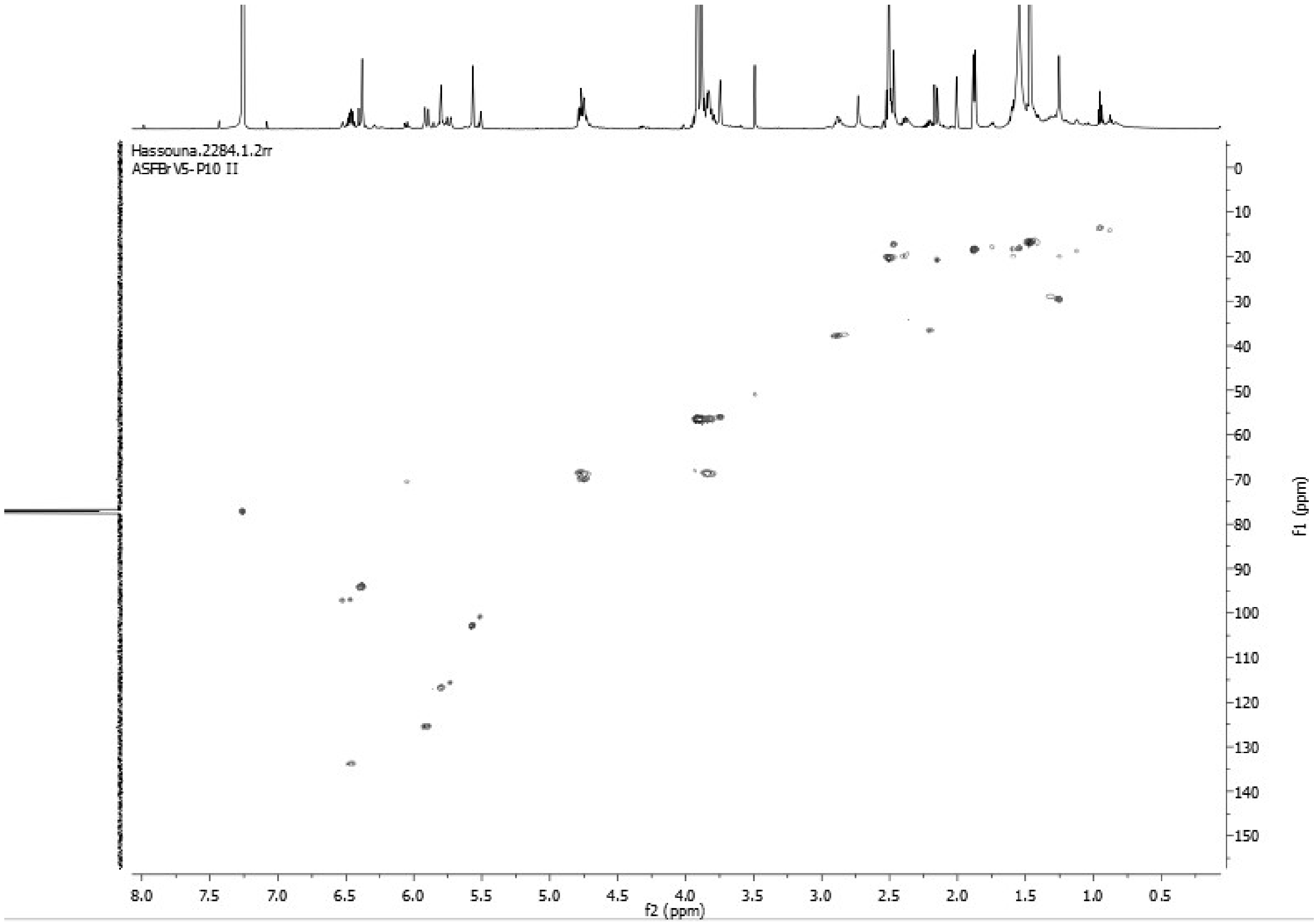

Figure S28. HMBC (600 MHz/150 MHz, $CDCl_3$) spectrum of compound **8**.

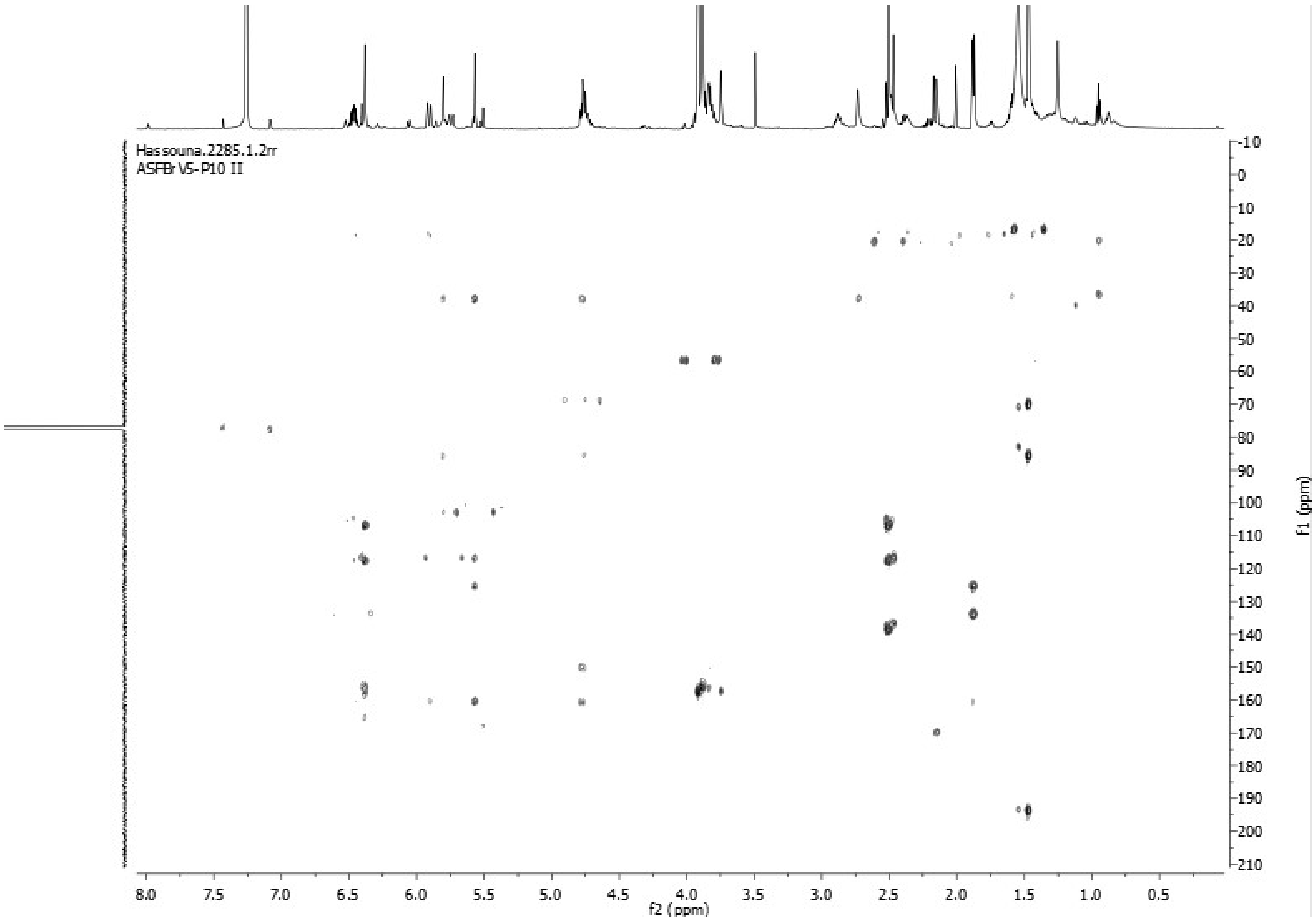

Figure S29. ROESY (600 MHz, $CDCl_3$) spectrum of compound **8**.

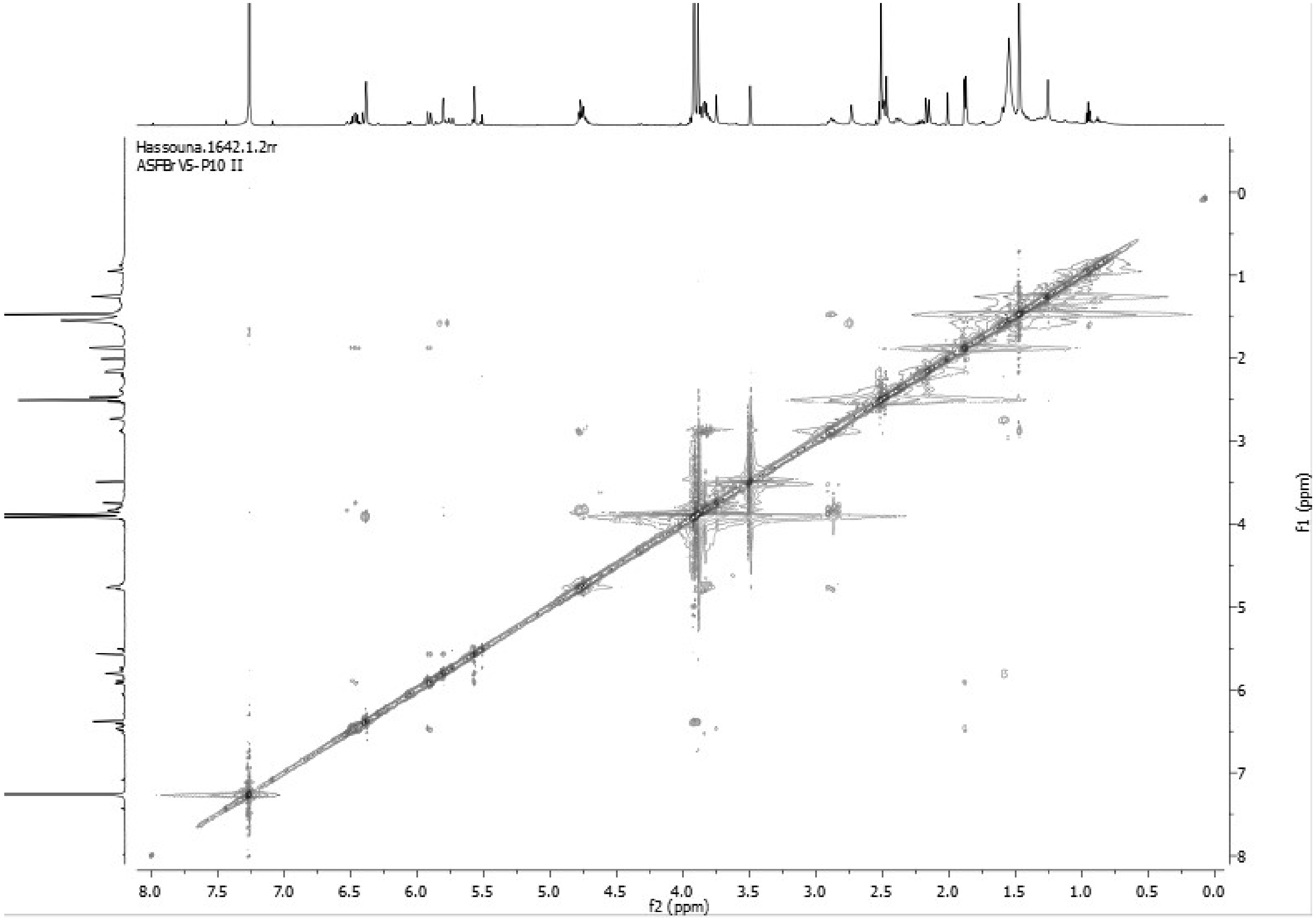

Figure S30. UV spectrum of compound **9**.

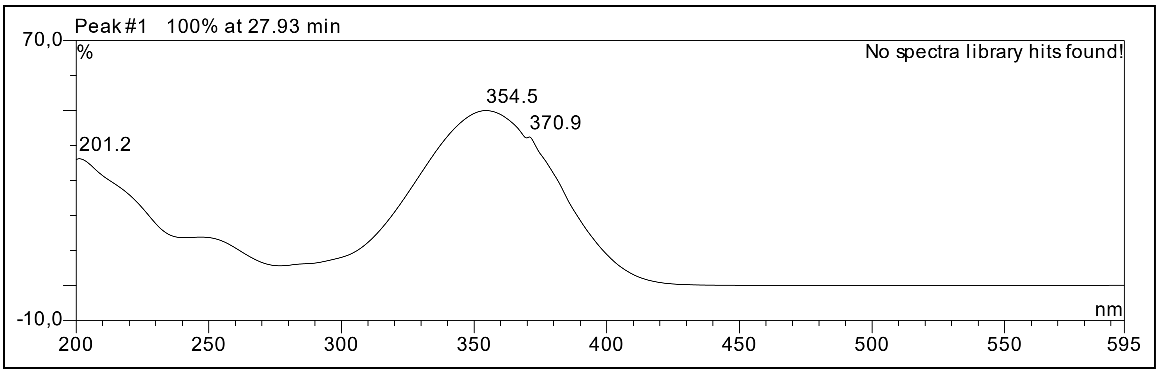

Figure S31. HRESIMS of compound **9**.

Mass Spectrum SmartFormula Report

Analysis Info

Analysis Name	D:\Data\Spektren 2019\PRO19HR000029.d	Acquisition Date	3/28/2019 8:30:17 AM
Method	tune_low_new.m	Operator	Peter Tommes
Sample Name	D. Hassouna ASFBr-V5-P9 (CH3OH)	Instrument	maXis 288882.20213
Comment			

Acquisition Parameter

Source Type	ESI	Ion Polarity	Positive	Set Nebulizer	0.3 Bar
Focus	Not active	Set Capillary	4000 V	Set Dry Heater	180 °C
Scan Begin	50 m/z	Set End Plate Offset	-500 V	Set Dry Gas	4.0 l/min
Scan End	1500 m/z	Set Collision Cell RF	600.0 Vpp	Set Divert Valve	Source

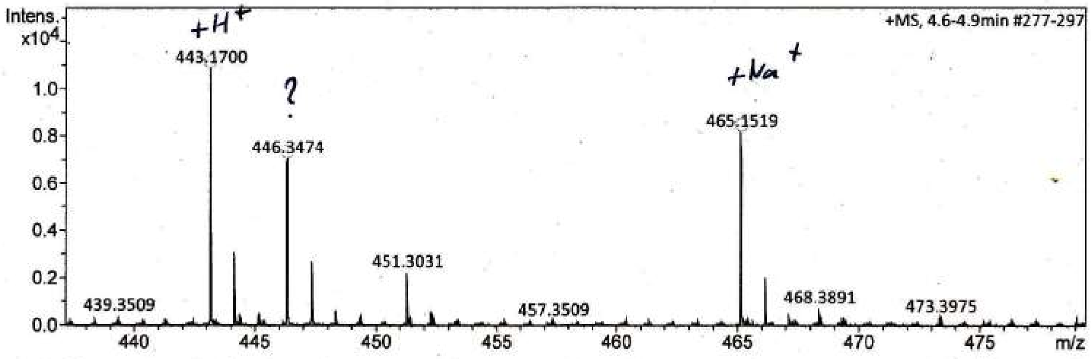

Meas. m/z	#	Ion Formula	m/z	err [ppm]	mSigma	# mSigma	Score	rdb	e⁻ Conf	N-Rule
443.1700	1	C24H27O8	443.1700	0.0	9.7	1	100.00	11.5	even	ok
446.3474	1	C24H48NO6	446.3476	0.6	66.1	1	100.00	1.5	even	ok
	2	C23H45N5NaO2	446.3465	-1.8	66.8	2	75.02	3.5	even	ok
465.1519	1	C24H26NaO8	465.1520	0.3	10.2	1	100.00	11.5	even	ok

Figure S32. ^{1}H NMR (700 MHz, $CDCl_3$) spectrum of compound **9**.

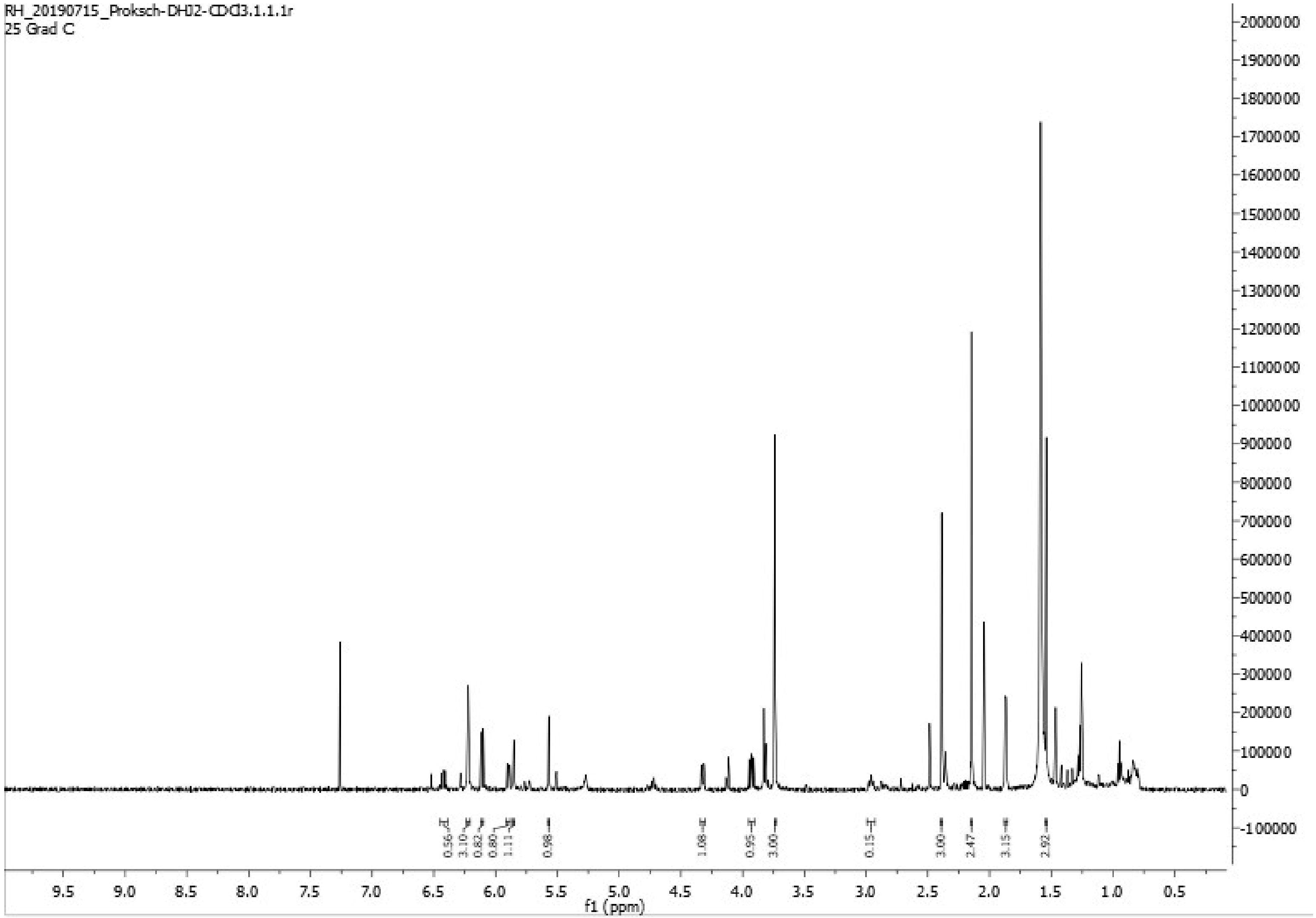

Figure S33. ^{13}C NMR (175 MHz, $CDCl_3$) spectrum of compound **9**.

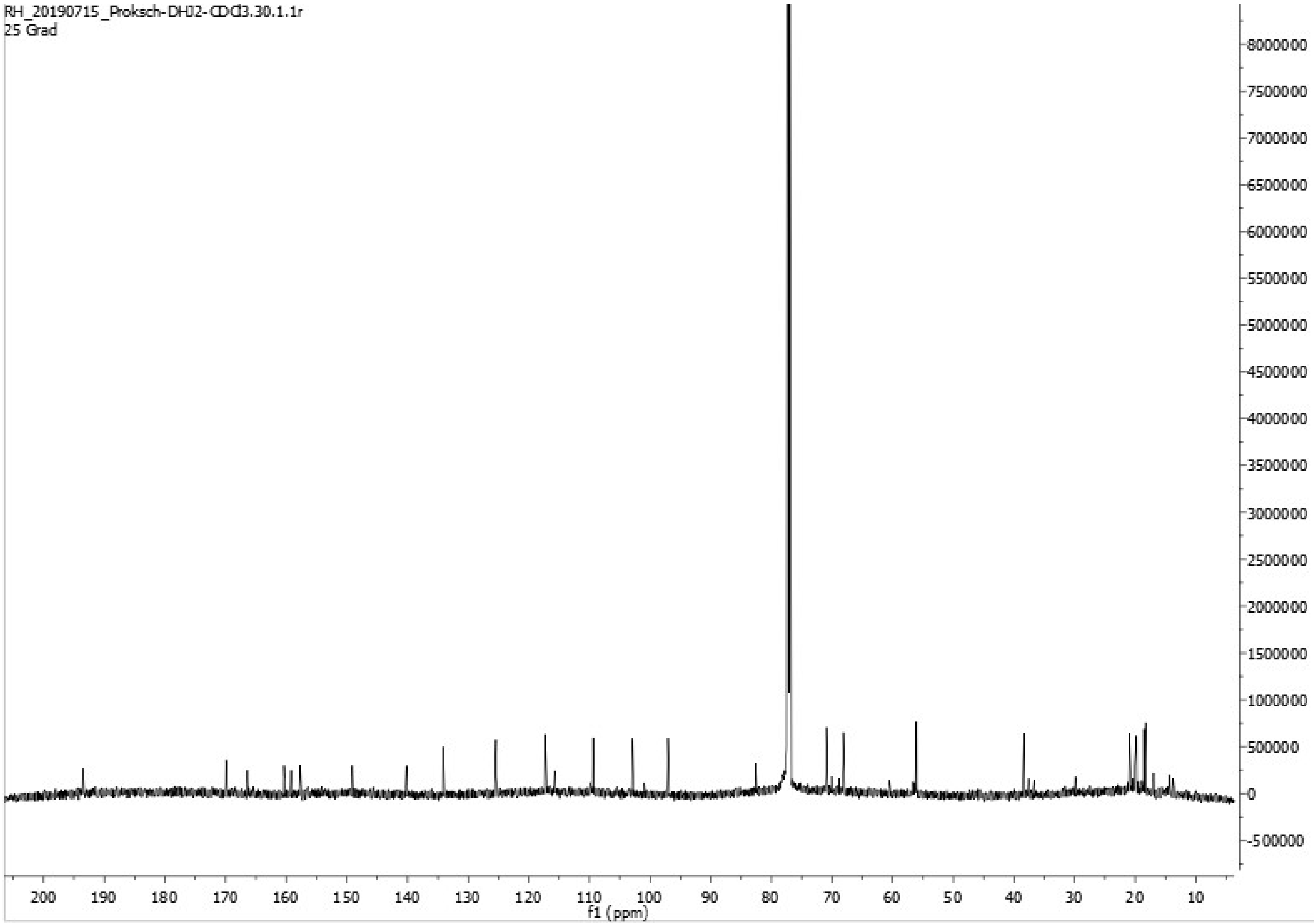

Figure S34. COSY (700 MHz, $CDCl_3$) spectrum of compound **9**.

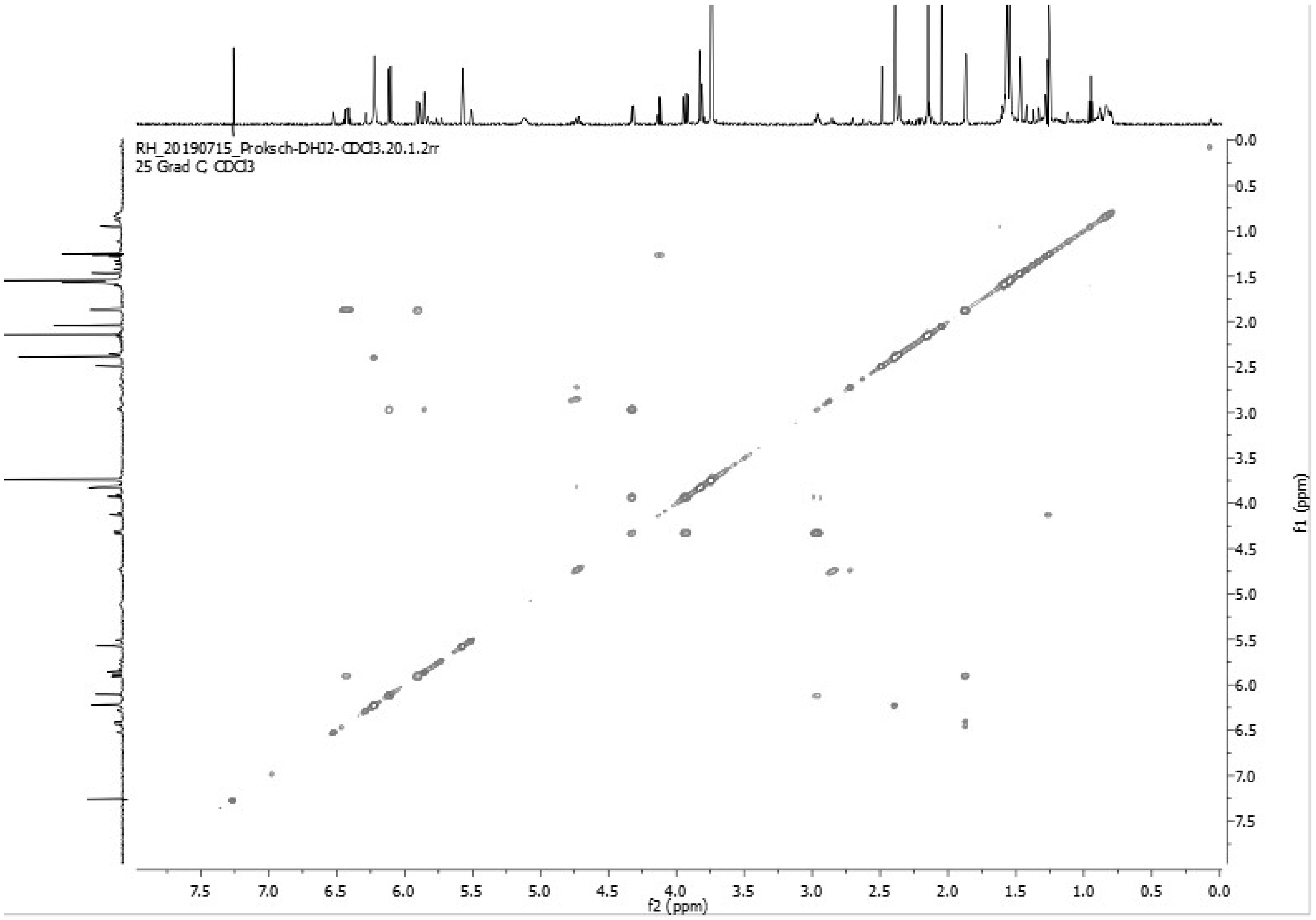

Figure S35. HSQC (700 MHz/175 MHz, $CDCl_3$) spectrum of compound **9**.

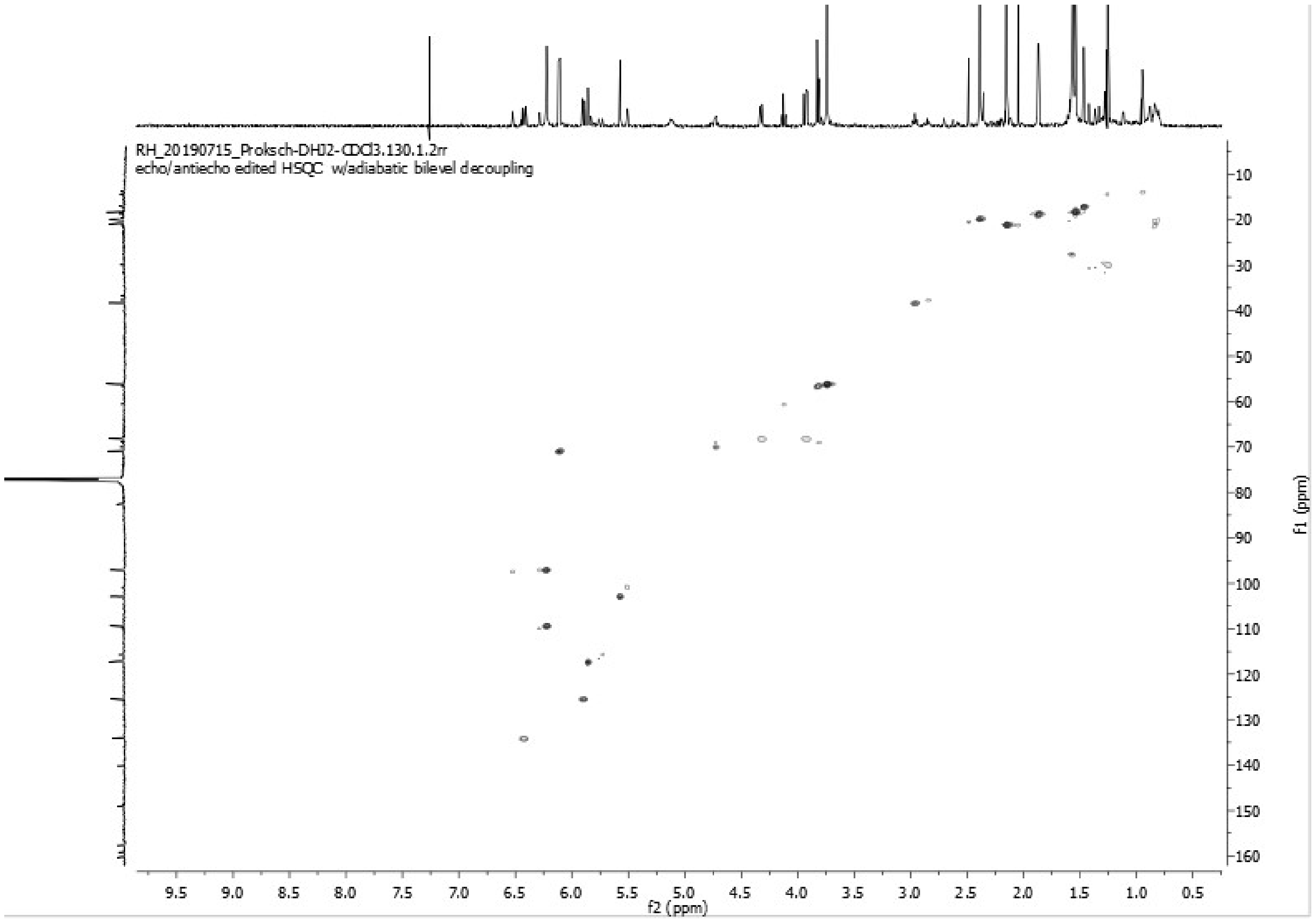

Figure S36. HMBC (700 MHz/175 MHz, $CDCl_3$) spectrum of compound **9**.

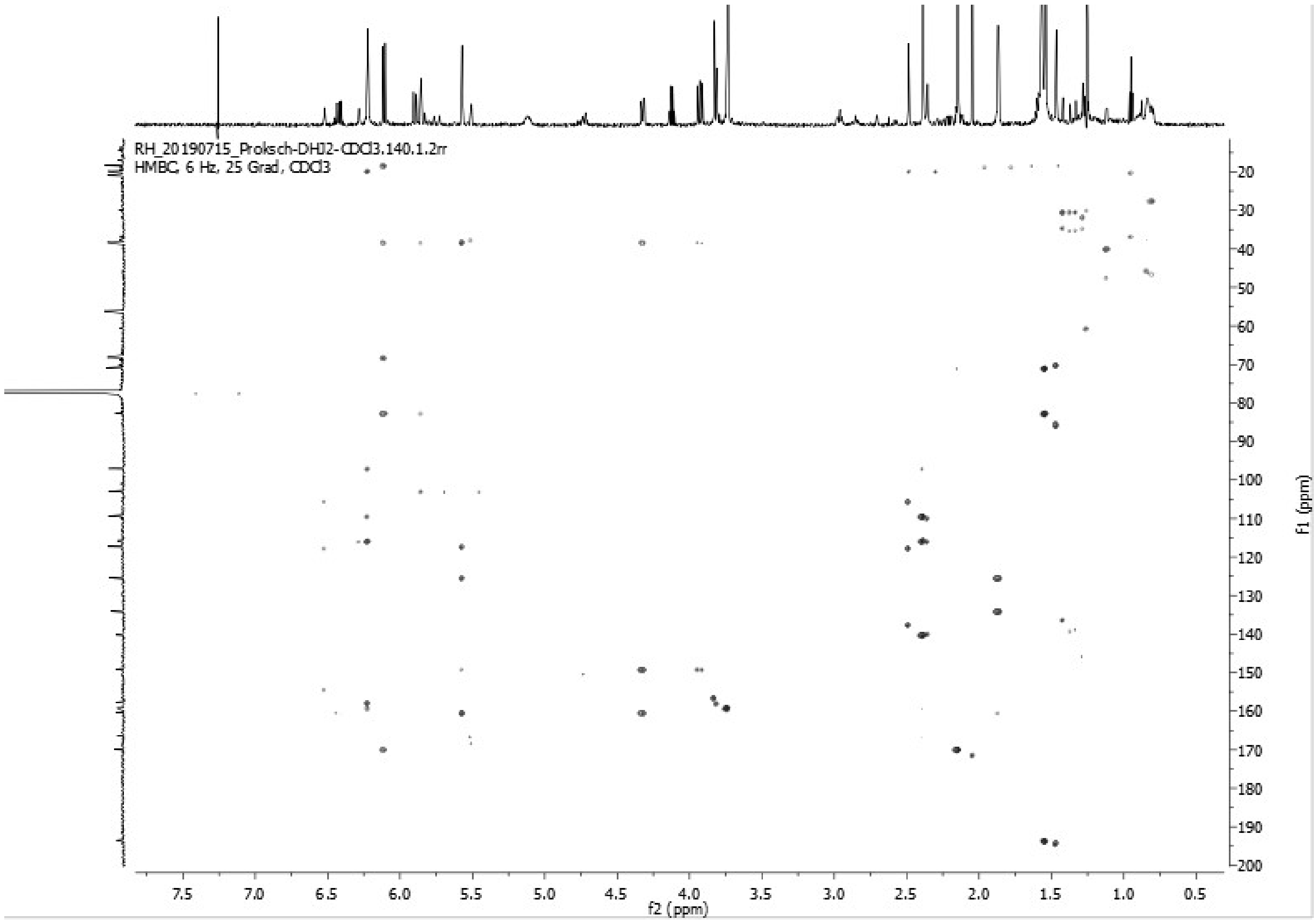

Figure S37. ROESY (700 MHz, $CDCl_3$) spectrum of compound **9**.

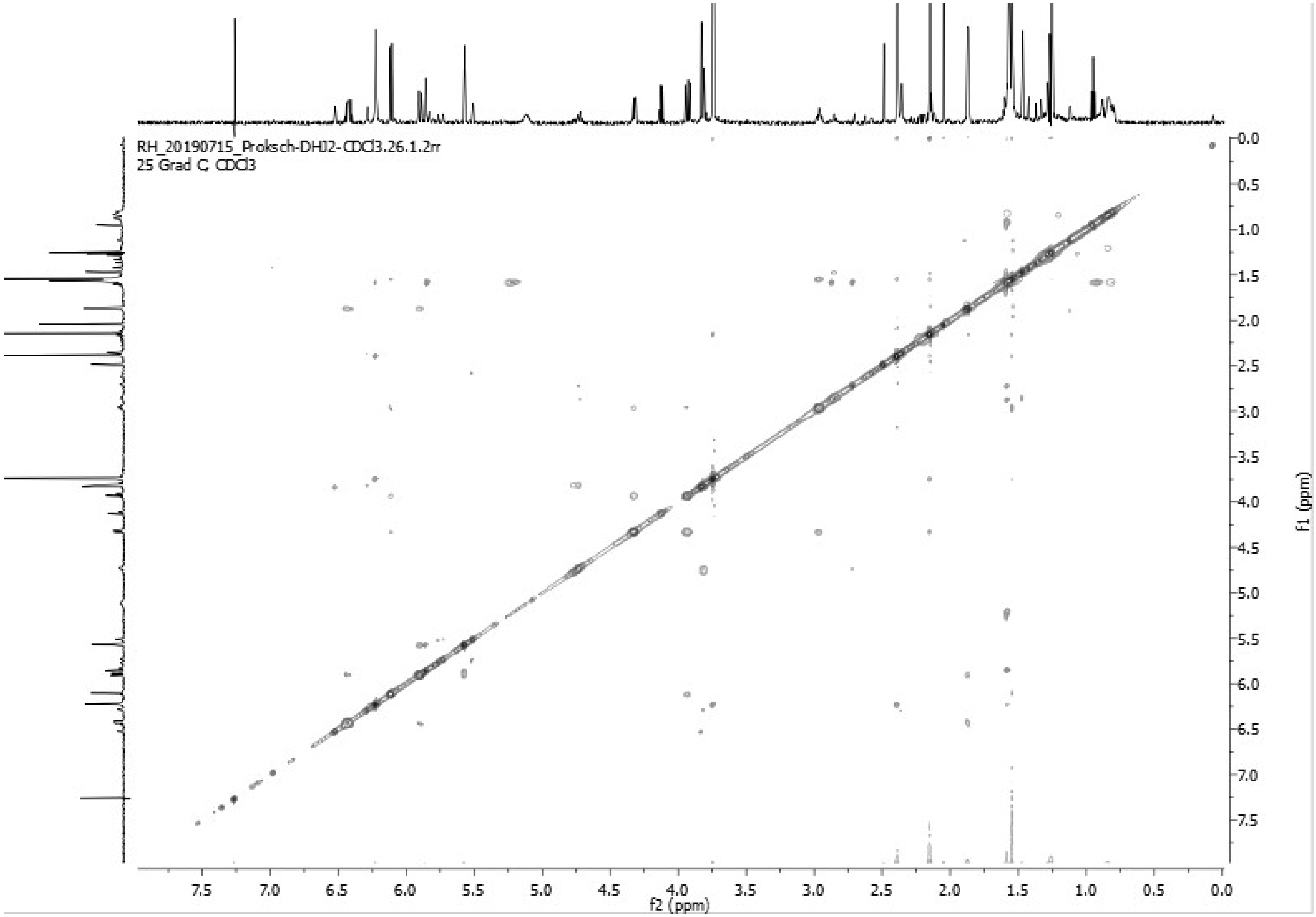

Figure S38. Section of the packing diagram over an extended unit cell of compound **5** (50% thermal elipsoids) determined by single-crystal x-ray diffraction. Hydrogen bonds are shown in dashed yellow lines.

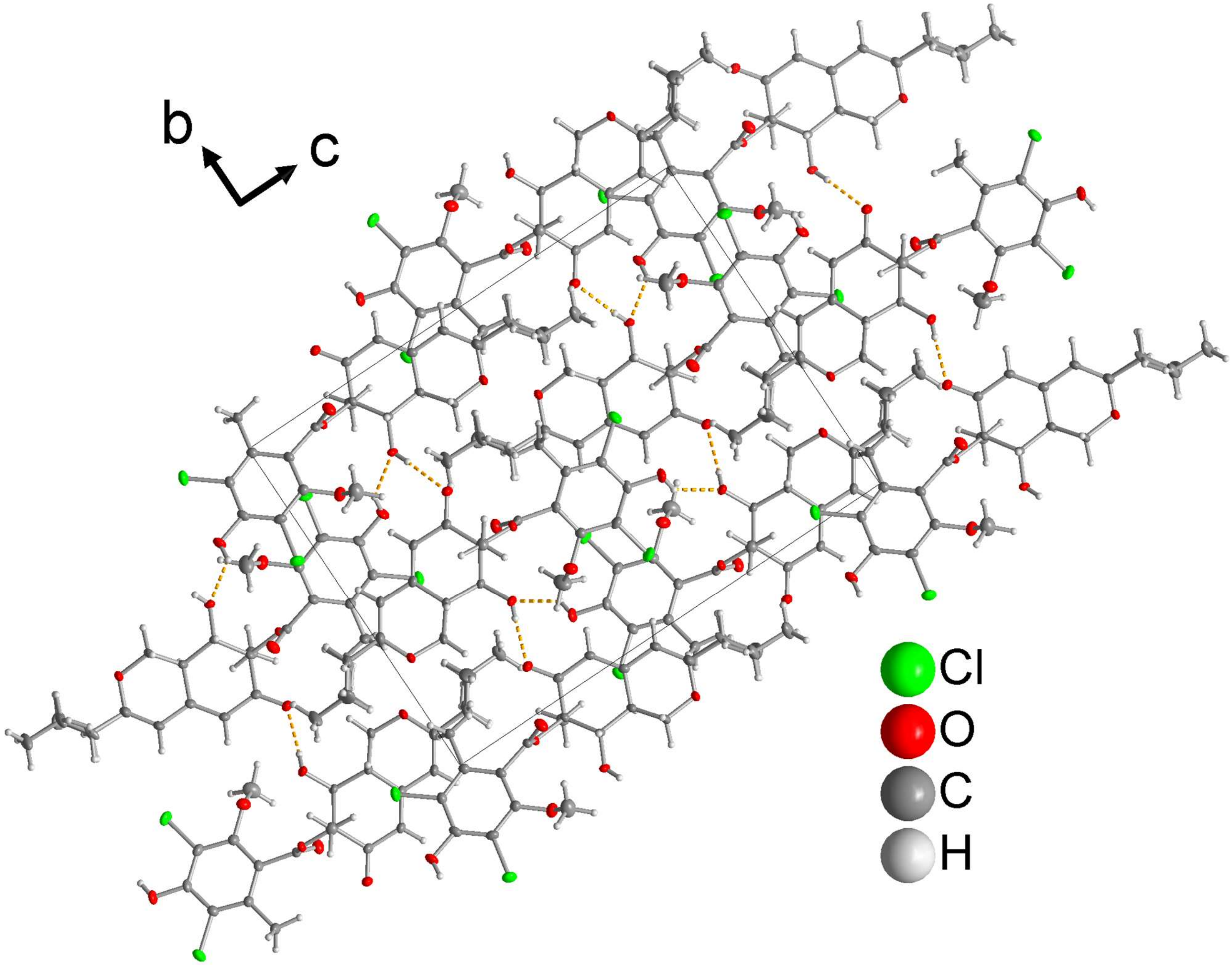

Figure S39. NFκB inhibitory potential of the compounds **1**, **3, 4**, **5**, **6**, **7**, **8**, **9** and **11**. Quantification of NFκB-dependent luciferase activity was performed by NFκB inhibition assay. In short, NFκB-MDA-MB-231 cells were pre-treated with the twofold serial diluted compound starting with 400 µM (**3**, **6**, **11**) or 200 µM to 0.78 µM (**1**, **4**, **5**, **7**, **9**, **11**) or left untreated (**Neg**). TNFα incubation induced NFκB activation, with untreated cells showing the maximal NFκB activity (**TNFα**). For RLU normalization, the RLU at the lowest concentration (0.78 µM) in each individual experiment was set as 100 %. Each data point represents the mean of at least three independent experiments. After the logarithmic transformation of the compound concentration in molar, nonlinear regression analysis without curve fitting was applied for data illustration using GraphPad Prism (GraphPad Software, San Diego, USA; Version 8.1.2). (M) Compound concentration in molar.

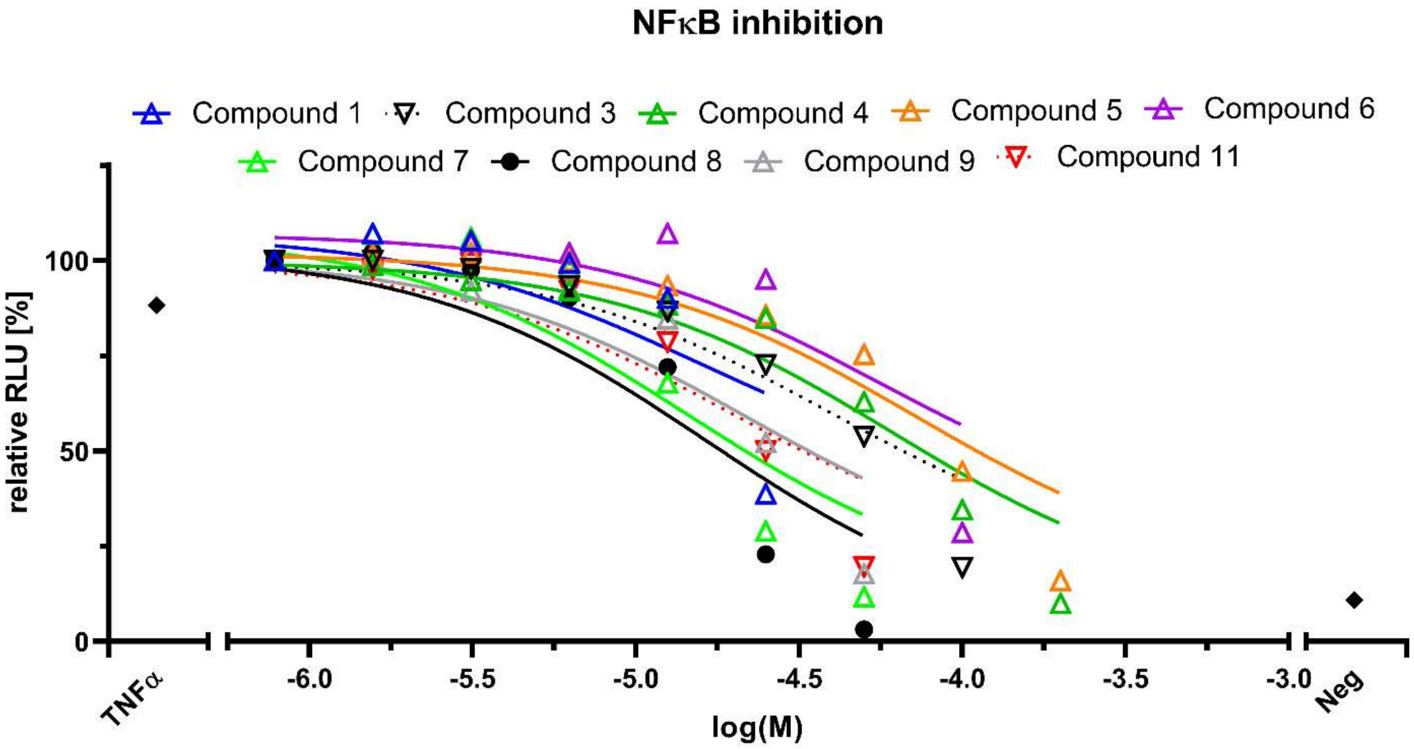

Figure S40. Potency in NFκB inhibition versus cytotoxicity. The scatterplot displays the pIC50 for the cell viability plotted against the pIC50 for the NFκB inhibition assay for the compounds **1**, **3**, **4**, **5**, **6**, **7**, **8**, **9** and **11**. The pIC_{50} value is determined as the negative decadic logarithm of the IC_{50}, which was calculated by nonlinear regression analysis without curve fitting using GraphPad Prism (GraphPad Software, San Diego, USA; Version 8.1.2). Compounds above the dotted line show greater potency in NFκB inhibition vs. cell viability and vice versa.

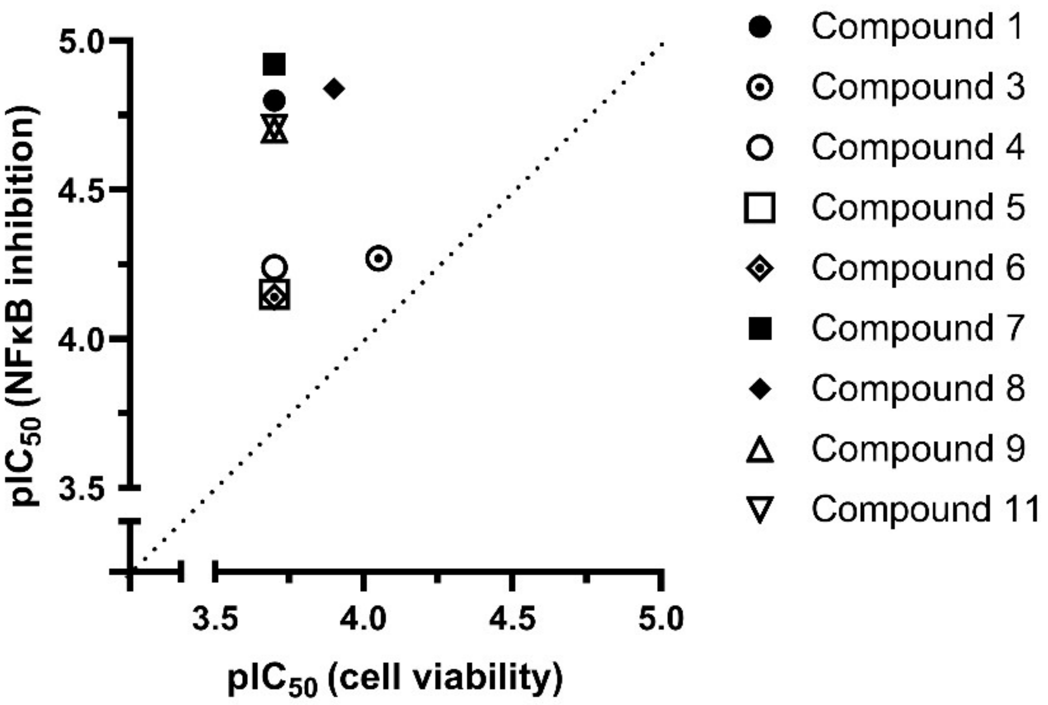

Table S1: Crystal data for compound **5**.

CCDC number	1976223
Empirical formula	$C_{22}H_{24}C_2O_7$
M [g mol^{-1}]	471.31
Crystal size [mm^3]	0.3 x 0.3 x 0.3
Temperature [K]	140
θ range [°] (completeness)	3.9 – 65.9 (0.99)
h; k; l range	±9; -14 – 16; -21 – 22
Crystal system	Orthorhombic
Space group	$P2_12_12_1$
a [Å]	8.1520(5)
b [Å]	14.1088(9)
c [Å]	18.7346(12)
α [°]	90.0
β [°]	90.0
γ [°]	90.0
V [Å^3]	2154.8(2)
Z	4
Dcalc [mg m^{-3}]	1.453
μ (Cu Kα) [mm^{-1}]	3.08
F(000)	984
Max./min. transmission	0.753 / 0.600
Reflections collected	26106
Independent reflect. (Rint)	3718
Data/restraints/parameters	3718 / 311 / 0
Max./min. Δρ [eÅ^{-3}] [a]	-0.22 / 0.43
R_1/wR_2 [I>2σ(I)] [b]	0.026 / 0.069
R_1/wR_2 [all data] [b]	0.026 / 0.069
Goodness-of-fit on F^2 [c]	1.03
Flack parameter [d]	0.016(5)

[a] Largest difference peak and hole; [b] $R_1 = [\sum(||F_o| - |F_c||)/\sum|F_o|]$; $wR_2 = [\sum[w(F_o^2 - F_c^2)^2]/\sum[w(F_o^2)^2]]^{1/2}$; [c] Goodness-of-fit = $[\sum[w(F_o^2 - F_c^2)^2]/(n - p)]^{1/2}$; [d] Absolute structure parameter.

Table S2. Fractional atomic coordinates and isotropic or equivalent isotropic displacement parameters ($Å^2$) for compound **5**.

	x	*y*	*z*	U_{iso}*/U_{eq}	Occ. (<1)
Cl1	0.24321 (8)	0.81549 (4)	1.04217 (3)	0.03219 (17)	
O1	0.5589 (4)	0.69409 (13)	0.53721 (10)	0.0493 (7)	
O1"	0.2929 (2)	0.53104 (14)	0.82263 (9)	0.0280 (4)	
C1"	0.3988 (3)	0.55041 (16)	0.86460 (12)	0.0197 (5)	
C1A	0.5101 (5)	0.70018 (19)	0.61155 (13)	0.0388 (8)	
H1AA	0.389878	0.691728	0.615360	0.047*	
H1AB	0.537897	0.763740	0.630398	0.047*	
Cl2	0.39219 (8)	0.46808 (5)	1.13798 (3)	0.02943 (16)	
O2	0.5498 (2)	0.38313 (11)	0.78063 (9)	0.0257 (4)	
O2"	0.3218 (2)	0.73236 (13)	0.89865 (10)	0.0303 (4)	
C2"	0.3698 (3)	0.57644 (17)	0.94185 (12)	0.0205 (5)	
O3	0.5598 (2)	0.55473 (11)	0.84990 (8)	0.0181 (3)	
O3"	0.2951 (2)	0.66247 (14)	1.15017 (9)	0.0262 (4)	
C3	0.5456 (5)	0.6063 (2)	0.50865 (14)	0.0372 (7)	
C3"	0.3220 (3)	0.66943 (17)	0.95431 (13)	0.0221 (5)	
C3'	0.4783 (5)	0.6862 (3)	0.31316 (14)	0.0468 (9)	
H3'A	0.402981	0.733835	0.293872	0.056*	
H3'B	0.451852	0.623986	0.292861	0.056*	
H3'C	0.591316	0.703135	0.300684	0.056*	
H3"O	0.245 (5)	0.711 (3)	1.155 (2)	0.045 (10)*	
O4	0.6083 (2)	0.71764 (12)	0.76564 (9)	0.0224 (4)	
C4	0.5357 (3)	0.52728 (18)	0.54849 (12)	0.0253 (5)	
H4	0.514439	0.468460	0.525616	0.030*	
C4"	0.2934 (3)	0.69915 (17)	1.02408 (14)	0.0226 (5)	
C4A	0.5564 (3)	0.52915 (17)	0.62462 (12)	0.0193 (5)	
H4O	0.557 (5)	0.760 (3)	0.752 (2)	0.048 (11)*	
C5	0.5467 (3)	0.45072 (16)	0.66601 (12)	0.0198 (5)	
H5	0.528414	0.391237	0.643617	0.024*	
C5"	0.3139 (3)	0.63701 (18)	1.08111 (12)	0.0197 (5)	

C6	0.5628 (3)	0.45355 (16)	0.74267 (12)	0.0182 (5)	
C6"	0.3654 (3)	0.54475 (17)	1.06651 (12)	0.0198 (5)	
C7	0.6131 (3)	0.54889 (16)	0.77595 (11)	0.0179 (5)	
C7"	0.3956 (3)	0.51319 (16)	0.99741 (12)	0.0203 (5)	
C8	0.5449 (3)	0.63334 (15)	0.73403 (12)	0.0175 (5)	
H8	0.422487	0.633599	0.737541	0.021*	
C8"	0.1640 (4)	0.7509 (2)	0.86995 (18)	0.0431 (7)	
H8"A	0.172803	0.799089	0.832466	0.065*	
H8"B	0.091529	0.773985	0.907890	0.065*	
H8"C	0.118724	0.692472	0.849679	0.065*	
C8A	0.5954 (3)	0.62553 (16)	0.65544 (12)	0.0218 (5)	
H8A	0.716405	0.636047	0.651994	0.026*	
C9	0.7995 (3)	0.55032 (18)	0.78099 (13)	0.0242 (5)	
H9A	0.846684	0.543759	0.733123	0.036*	
H9B	0.836613	0.497662	0.811049	0.036*	
H9C	0.835308	0.610455	0.802039	0.036*	
C9"	0.4537 (4)	0.41479 (18)	0.98344 (13)	0.0278 (6)	
H9"A	0.458174	0.403747	0.931836	0.042*	
H9"B	0.377677	0.369501	1.005353	0.042*	
H9"C	0.563290	0.406388	1.003953	0.042*	
C1'A	0.5597 (12)	0.6085 (3)	0.42905 (19)	0.0314 (18)	0.775 (19)
H1'A	0.676302	0.618190	0.416185	0.038*	0.775 (19)
H1'B	0.526074	0.545947	0.410056	0.038*	0.775 (19)
C2'A	0.4610 (8)	0.6823 (5)	0.3940 (3)	0.0348 (14)	0.775 (19)
H2'A	0.492628	0.744633	0.413960	0.042*	0.775 (19)
H2'B	0.344101	0.671564	0.405934	0.042*	0.775 (19)
C1'B	0.467 (3)	0.6112 (11)	0.4296 (7)	0.027 (4)	0.225 (19)
H1'C	0.479037	0.549208	0.405513	0.033*	0.225 (19)
H1'D	0.348780	0.626511	0.432761	0.033*	0.225 (19)
C2'B	0.553 (7)	0.6847 (19)	0.3890 (14)	0.087 (13)	0.225 (19)
H2'C	0.671573	0.670387	0.386669	0.104*	0.225 (19)
H2'D	0.538411	0.747193	0.412247	0.104*	0.225 (19)

Table S3. Atomic displacement parameters (Å^2) for compound **5**.

	U^{11}	U^{22}	U^{33}	U^{12}	U^{13}	U^{23}
Cl1	0.0442 (4)	0.0200 (3)	0.0323 (3)	0.0081 (3)	0.0045 (3)	-0.0070 (2)
O1	0.120 (2)	0.0177 (9)	0.0104 (8)	-0.0031 (12)	0.0016 (11)	0.0000 (7)
O1"	0.0299 (9)	0.0345 (10)	0.0196 (8)	-0.0048 (8)	0.0009 (7)	-0.0044 (8)
C1"	0.0300 (12)	0.0145 (11)	0.0147 (10)	-0.0003 (10)	0.0004 (10)	-0.0010 (9)
C1A	0.088 (2)	0.0173 (13)	0.0114 (11)	0.0047 (14)	-0.0014 (13)	-0.0024 (10)
Cl2	0.0390 (3)	0.0322 (3)	0.0171 (3)	0.0051 (3)	-0.0005 (2)	0.0059 (2)
O2	0.0435 (10)	0.0141 (8)	0.0197 (8)	-0.0005 (7)	-0.0004 (8)	0.0014 (7)
O2"	0.0374 (10)	0.0263 (10)	0.0271 (10)	0.0031 (8)	0.0031 (8)	0.0060 (8)
C2"	0.0271 (11)	0.0190 (11)	0.0155 (11)	-0.0006 (9)	0.0041 (10)	-0.0026 (9)
O3	0.0259 (8)	0.0174 (8)	0.0110 (7)	0.0009 (6)	0.0014 (6)	-0.0010 (6)
O3"	0.0345 (10)	0.0285 (10)	0.0158 (9)	0.0020 (8)	0.0052 (7)	-0.0067 (7)
C3	0.073 (2)	0.0224 (13)	0.0163 (12)	0.0002 (14)	0.0015 (13)	-0.0033 (10)
C3"	0.0278 (12)	0.0189 (12)	0.0194 (12)	0.0008 (10)	0.0027 (10)	0.0010 (10)
C3'	0.074 (2)	0.0469 (19)	0.0195 (14)	0.0284 (17)	-0.0023 (14)	0.0035 (13)
O4	0.0379 (9)	0.0118 (8)	0.0175 (8)	0.0005 (8)	-0.0054 (7)	-0.0028 (7)
C4	0.0429 (14)	0.0179 (12)	0.0152 (11)	0.0005 (11)	0.0006 (10)	-0.0049 (9)
C4"	0.0264 (12)	0.0159 (11)	0.0255 (12)	0.0019 (9)	0.0045 (10)	-0.0049 (10)
C4A	0.0237 (11)	0.0166 (11)	0.0175 (11)	0.0009 (9)	0.0029 (9)	-0.0036 (9)
C5	0.0282 (11)	0.0142 (11)	0.0171 (11)	0.0004 (10)	0.0015 (9)	-0.0036 (9)
C5"	0.0197 (10)	0.0243 (12)	0.0152 (11)	-0.0002 (9)	0.0035 (9)	-0.0053 (10)
C6	0.0219 (11)	0.0144 (11)	0.0182 (11)	0.0023 (9)	0.0026 (9)	-0.0016 (9)
C6"	0.0218 (11)	0.0215 (12)	0.0162 (11)	0.0013 (9)	0.0009 (9)	0.0020 (9)
C7	0.0265 (11)	0.0155 (11)	0.0116 (10)	0.0013 (9)	0.0013 (9)	-0.0022 (9)
C7"	0.0250 (11)	0.0179 (11)	0.0180 (11)	0.0008 (9)	0.0023 (9)	-0.0013 (9)
C8	0.0263 (11)	0.0121 (10)	0.0143 (10)	-0.0005 (9)	-0.0004 (9)	-0.0028 (9)
C8"	0.0405 (16)	0.0493 (18)	0.0396 (17)	0.0110 (14)	0.0018 (14)	0.0076 (14)
C8A	0.0376 (13)	0.0140 (11)	0.0138 (11)	-0.0037 (10)	0.0030 (10)	-0.0021 (9)
C9	0.0245 (12)	0.0245 (12)	0.0235 (12)	0.0019 (10)	-0.0018 (10)	-0.0021 (10)
C9"	0.0462 (15)	0.0183 (12)	0.0190 (12)	0.0030 (12)	0.0069 (11)	-0.0019 (10)
C1'A	0.054 (5)	0.0257 (19)	0.0148 (18)	0.009 (2)	0.000 (2)	-0.0013 (13)
C2'A	0.049 (3)	0.041 (3)	0.014 (2)	0.014 (3)	-0.002 (2)	-0.0002 (19)
C1'B	0.013 (9)	0.053 (9)	0.016 (6)	-0.002 (6)	-0.002 (6)	-0.013 (6)
C2'B	0.18 (4)	0.040 (11)	0.038 (11)	-0.02 (2)	0.04 (2)	0.009 (9)

Table S4. Geometric parameters (Å, º) for compound **5**.

Cl1—C4"	1.725 (2)	C4A—C5	1.354 (3)
O1—C3	1.353 (3)	C4A—C8A	1.511 (3)
O1—C1A	1.451 (3)	C5—C6	1.443 (3)
O1"—C1"	1.199 (3)	C5—H5	0.9500
C1"—O3	1.342 (3)	C5"—C6"	1.395 (4)
C1"—C2"	1.512 (3)	C6—C7	1.538 (3)
C1A—C8A	1.506 (4)	C6"—C7"	1.391 (3)
C1A—H1AA	0.9900	C7—C9	1.523 (3)
C1A—H1AB	0.9900	C7—C8	1.532 (3)
Cl2—C6"	1.735 (2)	C7"—C9"	1.490 (3)
O2—C6	1.226 (3)	C8—C8A	1.533 (3)
O2"—C3"	1.370 (3)	C8—H8	1.0000
O2"—C8"	1.419 (4)	C8"—H8"A	0.9800
C2"—C7"	1.387 (3)	C8"—H8"B	0.9800
C2"—C3"	1.388 (3)	C8"—H8"C	0.9800
O3—C7	1.454 (3)	C8A—H8A	1.0000
O3"—C5"	1.351 (3)	C9—H9A	0.9800
O3"—H3"O	0.80 (4)	C9—H9B	0.9800
C3—C4	1.344 (4)	C9—H9C	0.9800
C3—C1'A	1.496 (4)	C9"—H9"A	0.9800
C3—C1'B	1.614 (14)	C9"—H9"B	0.9800
C3"—C4"	1.392 (4)	C9"—H9"C	0.9800
C3'—C2'A	1.523 (6)	C1'A—C2'A	1.470 (8)
C3'—C2'B	1.55 (4)	C1'A—H1'A	0.9900
C3'—H3'A	0.9800	C1'A—H1'B	0.9900
C3'—H3'B	0.9800	C2'A—H2'A	0.9900
C3'—H3'C	0.9800	C2'A—H2'B	0.9900
O4—C8	1.426 (3)	C1'B—C2'B	1.46 (4)
O4—H4O	0.77 (4)	C1'B—H1'C	0.9900
C4—C4A	1.436 (3)	C1'B—H1'D	0.9900
C4—H4	0.9500	C2'B—H2'C	0.9900
C4"—C5"	1.392 (4)	C2'B—H2'D	0.9900
C3—O1—C1A	114.3 (2)	C2"—C7"—C6"	117.7 (2)
O1"—C1"—O3	125.4 (2)	C2"—C7"—C9"	121.1 (2)
O1"—C1"—C2"	124.8 (2)	C6"—C7"—C9"	121.2 (2)

O3—C1"—C2"	109.79 (19)	O4—C8—C7	107.72 (18)
O1—C1A—C8A	110.9 (2)	O4—C8—C8A	111.21 (19)
O1—C1A—H1AA	109.5	C7—C8—C8A	109.82 (18)
C8A—C1A—H1AA	109.5	O4—C8—H8	109.3
O1—C1A—H1AB	109.5	C7—C8—H8	109.3
C8A—C1A—H1AB	109.5	C8A—C8—H8	109.3
H1AA—C1A—H1AB	108.1	O2"—C8"—H8"A	109.5
C3"—O2"—C8"	114.1 (2)	O2"—C8"—H8"B	109.5
C7"—C2"—C3"	121.6 (2)	H8"A—C8"—H8"B	109.5
C7"—C2"—C1"	122.6 (2)	O2"—C8"—H8"C	109.5
C3"—C2"—C1"	115.8 (2)	H8"A—C8"—H8"C	109.5
C1"—O3—C7	119.03 (17)	H8"B—C8"—H8"C	109.5
C5"—O3"—H3"O	113 (3)	C1A—C8A—C4A	108.9 (2)
C4—C3—O1	123.0 (2)	C1A—C8A—C8	110.5 (2)
C4—C3—C1'A	125.1 (3)	C4A—C8A—C8	112.05 (19)
O1—C3—C1'A	111.7 (3)	C1A—C8A—H8A	108.4
C4—C3—C1'B	121.4 (6)	C4A—C8A—H8A	108.4
O1—C3—C1'B	110.8 (6)	C8—C8A—H8A	108.4
O2"—C3"—C2"	119.0 (2)	C7—C9—H9A	109.5
O2"—C3"—C4"	121.3 (2)	C7—C9—H9B	109.5
C2"—C3"—C4"	119.3 (2)	H9A—C9—H9B	109.5
C2'A—C3'—H3'A	109.5	C7—C9—H9C	109.5
C2'A—C3'—H3'B	109.5	H9A—C9—H9C	109.5
H3'A—C3'—H3'B	109.5	H9B—C9—H9C	109.5
C2'A—C3'—H3'C	109.5	C7"—C9"—H9"A	109.5
H3'A—C3'—H3'C	109.5	C7"—C9"—H9"B	109.5
H3'B—C3'—H3'C	109.5	H9"A—C9"—H9"B	109.5
C8—O4—H4O	108 (3)	C7"—C9"—H9"C	109.5
C3—C4—C4A	121.9 (2)	H9"A—C9"—H9"C	109.5
C3—C4—H4	119.0	H9"B—C9"—H9"C	109.5
C4A—C4—H4	119.0	C2'A—C1'A—C3	114.6 (5)
C5"—C4"—C3"	120.7 (2)	C2'A—C1'A—H1'A	108.6
C5"—C4"—Cl1	118.49 (19)	C3—C1'A—H1'A	108.6
C3"—C4"—Cl1	120.71 (19)	C2'A—C1'A—H1'B	108.6
C5—C4A—C4	123.2 (2)	C3—C1'A—H1'B	108.6
C5—C4A—C8A	121.9 (2)	H1'A—C1'A—H1'B	107.6
C4—C4A—C8A	114.9 (2)	C1'A—C2'A—C3'	114.7 (5)

C4A—C5—C6	122.8 (2)	C1'A—C2'A—H2'A	108.6
C4A—C5—H5	118.6	C3'—C2'A—H2'A	108.6
C6—C5—H5	118.6	C1'A—C2'A—H2'B	108.6
O3"—C5"—C4"	123.6 (2)	C3'—C2'A—H2'B	108.6
O3"—C5"—C6"	118.0 (2)	H2'A—C2'A—H2'B	107.6
C4"—C5"—C6"	118.3 (2)	C2'B—C1'B—C3	109 (2)
O2—C6—C5	123.2 (2)	C2'B—C1'B—H1'C	110.0
O2—C6—C7	119.8 (2)	C3—C1'B—H1'C	110.0
C5—C6—C7	116.9 (2)	C2'B—C1'B—H1'D	110.0
C7"—C6"—C5"	122.3 (2)	C3—C1'B—H1'D	110.0
C7"—C6"—Cl2	119.76 (18)	H1'C—C1'B—H1'D	108.4
C5"—C6"—Cl2	117.92 (18)	C1'B—C2'B—C3'	107 (3)
O3—C7—C9	103.80 (18)	C1'B—C2'B—H2'C	110.2
O3—C7—C8	109.62 (17)	C3'—C2'B—H2'C	110.2
C9—C7—C8	112.6 (2)	C1'B—C2'B—H2'D	110.2
O3—C7—C6	110.85 (18)	C3'—C2'B—H2'D	110.2
C9—C7—C6	107.64 (19)	H2'C—C2'B—H2'D	108.5
C8—C7—C6	112.06 (18)		

Table S5. Hydrogen-bond geometry (Å, º) for compound **5**.

D—H···*A*	*D*—H	H···*A*	*D*···*A*	*D*—H···*A*
O3"—H3"*O*···Cl1	0.80 (4)	2.58 (4)	2.989 (2)	113 (3)
O3"—H3"*O*···O4^{i}	0.80 (4)	2.11 (4)	2.769 (2)	139 (3)
O4—H4*O*···O2^{ii}	0.77 (4)	2.04 (4)	2.804 (2)	171 (4)

Symmetry codes: (i) *x*-1/2, -*y*+3/2, -*z*+2; (ii) -*x*+1, *y*+1/2, -*z*+3/2.
